# Association Between Cardiometabolic Multimorbidity and 15-year Mortality in the Asia Cohort Consortium

**DOI:** 10.2188/jea.JE20240362

**Published:** 2025-07-05

**Authors:** Sangjun Lee, Choonghyun Ahn, Sarah Krull Abe, Md Shafiur Rahman, Md Rashedul Islam, Eiko Saito, Seokyung An, Norie Sawada, Xiao-Ou Shu, Woon-Puay Koh, Hui Cai, Atsushi Hozawa, Seiki Kanemura, Chisato Nagata, San-Lin You, Daehee Kang, Rieko Kanehara, Yu-Tang Gao, Jian-Min Yuan, Wanqing Wen, Yumi Sugawara, Keiko Wada, Chien-Jen Chen, Keun-Young Yoo, Habibul Ahsan, Kee Seng Chia, Aesun Shin, Jeongseon Kim, Jung Eun Lee, Keitaro Matsuo, Nathaniel Rothman, You-Lin Qiao, Wei Zheng, Paolo Boffetta, Manami Inoue, Sue K. Park

**Affiliations:** 1 Department of Preventive Medicine, Seoul National University College of Medicine, Seoul, Korea; 2 Cancer Research Institute, Seoul National University, Seoul, Korea; 3 Integrated Major in Innovative Medical Science, Seoul National University Graduate School, Seoul, Korea; 4 Department of Orthopaedic Surgery, National Hospital Organization Sagamihara Hospital, Kanagawa, Japan; 5 Department of Orthopaedic Surgery, Tokyo University College of Medicine, Tokyo, Japan; 6 Division of Prevention, National Cancer Center Institute for Cancer Control, Tokyo, Japan; 7 Research Center for Child Mental Development, Hamamatsu University School of Medicine, Shizuoka, Japan; 8 Hitotsubashi Institute for Advanced Study, Hitotsubashi University, Tokyo, Japan; 9 Sustainable Society Design Center, Graduate School of Frontier Science, The University of Tokyo, Tokyo, Japan; 10 Division of Cohort Research, National Cancer Center Institute for Cancer Control, Tokyo, Japan; 11 Division of Epidemiology, Department of Medicine, Vanderbilt Epidemiology Center, Vanderbilt-Ingram Cancer Center, Vanderbilt University Medical Center, Nashville, TN, USA; 12 Healthy Longevity Translational Research Programme, Yong Loo Lin School of Medicine, National University of Singapore, Singapore, Singapore; 13 Singapore Institute for Clinical Sciences, Agency for Science Technology and Research (A*STAR), Singapore, Singapore; 14 Tohoku University Graduate School of Medicine, Miyagi Prefecture, Miyagi, Japan; 15 Department of Epidemiology and Preventive Medicine, Gifu University Graduate School of Medicine, Gifu, Japan; 16 School of Medicine & Big Data Research Center, Fu Jen Catholic University, Taipei, Taiwan; 17 Department of Epidemiology, Shanghai Cancer Institute, Shanghai, China; 18 Renji Hospital, Shanghai Jiaotong University School of Medicine, Shanghai, China; 19 Cancer Epidemiology and Prevention Program, University of Pittsburgh Medical Center (UPMC) Hillman Cancer Center, Pittsburgh, PA, USA; 20 Department of Epidemiology, School of Public Health, University of Pittsburgh, Pittsburgh, PA, USA; 21 Genomics Research Center, Academia Sinica, Taipei, Taiwan; 22 Veterans Health Service Medical Center, Seoul, Korea; 23 Department of Public Health Sciences, University of Chicago, IL, USA; 24 Saw Swee Hock School of Public Health, National University of Singapore, Singapore; 25 Graduate School of Cancer Science and Policy, National Cancer Center, Goyang, Korea; 26 Department of Food and Nutrition, Seoul National University, Seoul, Korea; 27 Division Cancer Epidemiology and Prevention, Aichi Cancer Center Research Institute, Nagoya, Japan; 28 Department of Cancer Epidemiology, Nagoya University Graduate School of Medicine, Nagoya, Japan; 29 Division of Cancer Epidemiology and Genetics, Occupational and Environmental Epidemiology Branch, National Cancer Institute, Bethesda, MD, USA; 30 School of Population Medicine and Public Health, Chinese Academy of Medical Sciences and Peking Union Medical College, Beijing, China; 31 Stony Brook Cancer Center, Stony Brook University, Stony Brook, NY, USA; 32 Department of Medical and Surgical Sciences, University of Bologna, Bologna, Italy

**Keywords:** cardiometabolic multimorbidity, prospective cohort study, Asian populations

## Abstract

**Background:**

Studies on the association between multimorbidity and mortality in large populations have mainly been conducted in European and North American populations. This study aimed to identify the association between cardiometabolic multimorbidity and all-cause and cardiovascular disease (CVD) mortality in the Asia Cohort Consortium.

**Methods:**

In this prospective cohort study, pooled analysis was performed to evaluate the association between cardiometabolic diseases (hypertension, diabetes, ischemic heart disease, and stroke), multimorbidity, and all-cause and CVD mortality, including premature mortality, among participants from 11 Asian cohort studies. Hazard ratios (HRs) and 95% confidence intervals (CIs) were estimated using Cox hazard regression.

**Results:**

A total of 483,532 participants were followed for a median of 14.3 years. Compared with participants without any disease, those with stroke and diabetes had higher age- and sex-adjusted HRs for all-cause mortality (HR 3.9; 95% CI, 3.28–4.56). Moreover, the age- and sex-adjusted HRs for CVD mortality were highest in participants with stroke, ischemic heart disease, and diabetes (HR 10.6; 95% CI, 6.16–18.25). These patterns remained consistent after additional adjustments for smoking status and body mass index. The risk of premature mortality followed similar trends but was more pronounced.

**Conclusion:**

These findings highlight the differential impacts of individual cardiometabolic diseases and their combinations on mortality risks. Stroke and diabetes were associated with the highest risks for all-cause and cardiovascular mortality, underscoring the need for targeted prevention and personalized management strategies tailored to these high-risk conditions in Asian populations.

## INTRODUCTION

Since 1950, rapid progress in life expectancy has markedly decreased the incidence of communicable, neonatal, and nutritional diseases, whereas the incidence of non-communicable diseases (NCDs) has increased.^1^ Tobacco smoking, high blood pressure, and high blood sugar levels are major risk factors for NCDs, including ischemic heart disease (IHD) and stroke. The prevalence of hypertension (HTN) and diabetes mellitus (DM) is rapidly increasing worldwide and causing early death in men and women, although both diseases are preventable.^1^ Mediated by the occurrence of atherosclerosis, HTN and DM are associated with an increased risk of IHD, stroke, and stroke mortality.^2^^,^^3^ Additionally, cardiometabolic diseases, such as HTN, DM, IHD, and stroke, are associated with an increased risk of long-term mortality.^2^^,^^4^^–^^6^

The prevalence of multimorbidity (defined as two or more chronic diseases in the same individual) has been rapidly increasing in many populations, owing to increases in life expectancy and NCD prevalence.^7^ The Emerging Risk Factors Collaboration (ERFC) reported that cardiometabolic multimorbidity due to DM, IHD, and stroke was associated with increased overall mortality and reduced life expectancy.^7^ The number of cardiometabolic diseases had a multiplicative effect on the increased risk of overall mortality. A limitation of the ERFC study was that HTN was excluded from multimorbidity evaluation.

The prevalence of HTN is higher in Asian populations than in European populations, the incidence of HTN-associated hemorrhagic stroke is higher in Asian populations, and the incidence of ischemic stroke is increasing.^8^ Therefore, further information is needed on cardiometabolic multimorbidity based on the four diseases (HTN, DM, IHD, and stroke) in Asian populations. Additionally, it is unclear whether mortality due to cardiometabolic diseases has a multiplicative effect in Asian populations, where the prevalence of risk factors and cardiometabolic diseases differs from those in Western populations.

Theoretically, individuals with multiple cardiovascular and metabolic diseases may have a higher mortality risk, and it is necessary to determine whether comorbidities have a synergistic or attenuating effect on mortality risk.^9^ We assumed that cardiometabolic multimorbidity was more strongly associated with an increased risk of CVD mortality than with overall mortality.^10^ Therefore, we analyzed the association between cardiometabolic multimorbidity, defined as the combination of four cardiometabolic diseases (HTN, DM, IHD, and stroke), and all-cause and CVD mortality in the Asia Cohort Consortium (ACC) framework, a collaborative effort to pool cohort studies performed in different Asian countries.^11^

## METHODS

### Study population

Approximately one million people participated in the ACC, which included 44 cohort studies from 10 Asian countries.^11^ Individual cohort studies provided information on various risk factors and medical and family history using questionnaires, anthropometric measurements, and laboratory tests, and they followed up on new cases and deaths according to the country-specific ascertainment system. Details of the ACC have been reported previously.^11^

Of these cohorts, 11 (*n* = 523,947) with baseline information on cardiometabolic multimorbidity and follow-up data on all-cause and CVD mortality were included in this analysis, of which six were from Japan, two were from China, and one cohort each was from South Korea, Taiwan, and Singapore. The participating cohorts were as follows: Japan Public Health Center-based prospective Study1 (JPHC1),^12^ Japan Public Health Center-based prospective Study2 (JPHC2),^12^ the Ohsaki National Health Insurance Cohort Study (Ohsaki),^13^ Miyagi Cohort Study (Miyagi),^13^ The Takayama Study (Takayama),^14^ Three-Prefecture Cohort Study, Miyagi (3pref. Miyagi),^15^ Community-based Cancer Screening Project (CBCSP),^16^ Korean Multi-center Cancer Cohort (KMCC),^17^ Singapore Chinese Health Study (SCHS),^18^ Shanghai Women’s Health Study (SWHS),^19^ Shanghai Men’s Health Study (SMHS).^20^

Of the 523,947 participants, 2,984 were excluded because of missing baseline information on sex (*n* = 114) and vital status (*n* = 2,870). A further 12,676 participants with missing data on weight (*n* = 1,167) or height (*n* = 11,509) were excluded. Furthermore, 124 participants were excluded because their follow-up period was negative, and 24,631 participants were excluded because of a missing disease history. Finally, 483,532 individuals were included in the analysis (Table 1).

**Table 1.** Selected characteristics of each cohort study participants in the Asia Cohort Consortium (ACC)

Men	Cohort	Study Entry	Person- years	Mean age	Mean BMI	HTN	DM	IHD	Stroke	All-cause		CVD	
			
	*N*	Year	Years	Years	kg/m^2^	*N* (%)	*N* (%)	*N* (%)	*N* (%)	*N*	CR	*N*	CR
			
**Japan**													
JPHC1	20,444	1990–1992	417,888.2	49.5	23.6	3,424 (16.7)	1,138 (5.6)	270 (1.3)	113 (0.6)	4,854	11.6	1,212	2.9
JPHC2	26,403	1992–1995	450,480.2	54.0	23.5	5,769 (21.8)	2,041 (7.7)	428 (1.6)	339 (1.3)	7,831	17.4	1,873	4.2
Ohsaki	23,000	1995	244,451.9	59.5	23.3	5,689 (24.7)	1,748 (7.6)	765 (3.3)	668 (2.9)	5,161	21.1	1,595	6.5
Miyagi	20,254	1990	336,756.1	51.6	23.6	3,783 (18.7)	1,097 (5.4)	319 (1.6)	207 (1.0)	3,538	10.5	614	1.8
Takayama	11,703	1992	154,770.6	55.2	22.5	2,812 (24.0)	868 (7.4)	639 (5.5)	237 (2.0)	2,675	17.3	824	5.3
3pref. Miyagi	13,297	1984	148,661.2	56.5	23.1	3,648 (27.4)	1,032 (7.8)	1,121 (8.4)	170 (1.3)	3,290	20.1	1,202	8.1

**Taiwan**													
CBCSP	11,930	1991–1992	178,438.4	48.0	24.0	766 (6.4)	333 (2.8)	244 (2.1)	88 (0.7)	1,833	10.3	366	2.1

**Korea**													
KMCC	6,651	1993–2005	85,302.9	53.1	23.1	605 (9.1)	304 (4.6)	119 (1.8)	110 (1.7)	1,667	19.5	287	3.4

**Singapore**													
SCHS	27,954	1993–1999	310,933.5	56.7	23.0	6,555 (23.4)	2,425 (8.7)	1,369 (4.9)	507 (1.2)	6,144	19.8	2,099	6.8

**China**													
SMHS	61,406	2001–2006	588,782.9	55.4	23.7	18,321 (29.8)	3,856 (6.3)	3,144 (5.1)	2,314 (3.8)	5,428	9.2	1,792	3.0



Women	Cohort	Study Entry	Person- years	Mean age	Mean BMI	HTN	DM	IHD	Stroke	All-cause		CVD	
			
	*N*	Year	Years	Years	kg/m^2^	*N* (%)	*N* (%)	*N* (%)	*N* (%)	*N*	CR	*N*	CR
			
**Japan**													
JPHC1	22,288	1990–1992	479,606.0	49.7	23.6	3,429 (15.4)	559 (2.5)	216 (1.0)	61 (0.3)	2,542	5.3	625	1.3
JPHC2	29,276	1992–1995	536,295.0	54.4	23.4	6,031 (20.6)	1,097 (3.7)	221 (0.8)	144 (0.5)	4,689	8.7	1,274	2.4
Ohsaki	24,710	1995	269,910.9	60.7	23.8	7,081 (28.7)	1,422 (5.8)	576 (2.3)	395 (1.6)	2,862	10.6	1,142	4.2
Miyagi	21,938	1990	359,406.6	52.3	23.8	4,453 (20.3)	702 (3.2)	215 (1.0)	112 (0.5)	1,739	4.8	749	2.1
Takayama	14,077	1992	196,250.5	56.0	22.0	2,964 (21.1)	467 (3.3)	694 (4.9)	148 (1.1)	2,247	11.4	884	4.5
3pref. Miyagi	16,257	1984	192,854.2	57.1	23.4	4,180 (25.7)	665 (4.1)	1,508 (9.3)	121 (0.7)	2,595	13.5	1,103	5.7

**Taiwan**													
CBCSP	11,768	1991–1992	182,804.9	46.6	24.1	657 (5.6)	262 (2.2)	201 (1.7)	30 (0.3)	916	5.0	188	1.0

**Korea**													
KMCC	9,961	1993–2005	143,115.2	53.5	23.9	1,271 (12.8)	465 (4.7)	224 (2.2)	119 (1.2)	1,374	9.6	418	2.9

**Singapore**													
SCHS	35,303	1993–1999	414,071.8	56.3	23.2	8,499 (24.1)	3,271 (9.3)	1,229 (3.5)	440 (1.2)	4,548	11.0	1,609	3.9

**China**													
SWHS	74,912	1996–2000	1,115,975.1	52.6	24.0	17,822 (23.8)	3,298 (4.4)	5,525 (7.4)	882 (1.2)	7,640	6.8	2,486	2.2

3pref Miyagi, Three-Prefecture Cohort Study, Miyagi; CBCSP, Community-based Cancer Screening Project; DM, diabetes mellitus; HTN, hypertension; IHD, ischemic heart diseases, including myocardial infarction; JPHC1, Japan Public Health Center-based prospective Study1; JPHC2, Japan Public Health Center-based prospective Study2; KMCC, Korean Multi-center Cancer Cohort; Miyagi, Miyagi Cohort study; Ohsaki, the Ohsaki National Health Insurance Cohort Study; SCHS, Singapore Chinese Health Study; SMHS, Shanghai Men’s Health Study; Takayama, The Takayama Study.

### Outcome assessment

The primary outcomes were all-cause mortality (except for S/T codes in international Classification of Diseases [ICD] 10th edition, which measured the disability of each injury diagnosis) and CVD mortality (ICD-9-codes 390–459; ICD-10-codes I00–I99). Premature mortality refers to death occurring before the average age of death within a population.^21^ Premature mortality was quantified by calculating the number of years of potential life lost (YPLL). The YPLL assesses age at death compared with the average life expectancy, estimating the average number of years an individual might have lived if they had not died of a specific disease at an earlier age.^22^ Based on the World Health Organization’s recommended upper limit for premature mortality, we used 70 years as the age for premature mortality.^23^ Additionally, we obtained all-cause and CVD mortality data for each continent from the Global Burden of Disease project, which provides the most comprehensive global estimate for mortality studies (eFigure 1 and eFigure 2).^24^ Based on the YPLL and mortality rate (per 100,000 population), a sensitivity analysis was performed using 65 years of age as the age limit for premature mortality in men.

### Assessment of exposure and potential confounders

Cardiometabolic multimorbidity was defined as a self-reported medical history of HTN, DM, IHD (including myocardial infarction [MI]), or stroke (all types) at the baseline.^25^^–^^27^ This definition was chosen to provide a comprehensive evaluation of the cumulative burden of diseases that not only predispose individuals to cardiovascular events but also represent clinical outcomes themselves. This approach aligns with prior studies, such as those by Prados-Torres et al (2014),^28^ which identified common multimorbidity patterns comprising HTN, DM, heart disease, and obesity, often linked to metabolic syndrome. Similarly, Canoy et al (2021) noted that cardiometabolic studies frequently include stroke and MI as part of the disease cluster when examining mortality risk.^29^ The participants were categorized into 16 mutually exclusive groups according to their baseline disease history (eTable 1). Information on age at cohort enrolment, sex, cigarette smoking status (never/ever), and body mass index (BMI) was collected from participants in the 11 cohorts using structured questionnaires and harmonized by the ACC coordinating center.

### Statistical analysis

Cox proportional hazard regression (CPH) models were used to estimate hazard ratios (HRs) and 95% confidence intervals (CIs). The equations are listed in eTable 1. Person-years were calculated by summing the follow-up time for each participant until the outcome or the end of the study period, whichever came first.

First, we estimated the association between the 16 categorical types of cardiometabolic multimorbidity and the risk of all-cause and CVD mortality, including premature mortality. We conducted the analyses by sequentially adding adjustment variables, starting from age at enrolment and sex to cigarette smoking, BMI, and cohorts. To address the potential influence of non-CVD mortality as a competing risk, we performed a competing risk analysis using the Fine-Gray sub-distribution hazard model.^30^ This method estimates sub-distribution hazard ratios (SHRs) and 95% CIs for CVD mortality while adjusting for age, cigarette smoking, BMI, and cohorts and accounting for the competing risk of non-CVD mortality.^30^ For premature mortality, CPH model were performed using the definition of age at death <75 years for both men and women. Sensitivity analyses were conducted excluding the first 2 and 5 years of follow-up to minimize the influence of possible reverse causation due to the presence of terminal disease at baseline in some participants. Second, stratified analyses were conducted according to sex, adjusting for age at enrolment, cigarette smoking status, and BMI. Third, we re-analyzed the data according to the ERFC and UK Biobank disease combination categories and compared the risk of all-cause and CVD mortality to the original estimates. Fourth, we also conducted additional analyses to distinguish between ischemic stroke (ICD-10 code: I63) and hemorrhagic stroke (ICD-10 codes: I60–I62) as separate outcomes, addressing the differential mortality risks associated with these subtypes. Fifth, we performed a sensitivity analysis using different definitions of MI instead of IHD, adjusted for age at enrolment, sex, cigarette smoking, and BMI. Sixth, the population attributable fraction (PAF) was estimated using Levin’s formula^31^ based on 1) the HRs of cardiometabolic and multimorbidity for all-cause and CVD mortality and 2) the age-standardized prevalence in the age group over 40 years for the year 2000 from the Eastern Asian population provided by World Population Prospects 2022.^32^ The 95% CIs of the PAFs were estimated using Monte Carlo simulation.^33^

All analyses were performed using the R software version 4.3.0 (R Foundation for Statistical Computing, Vienna, Austria) and SAS version 9.4 (SAS Institute, Cary, NC, USA).

## RESULTS

Participant characteristics are shown in Table 1. Of the 483,532 cohort members, 73,573 and 22,342 participants died from all causes and CVD, respectively, over a median follow-up period of 14.3 years. The mean age at baseline was 54.3 years, and 54.0% of participants were women. Of the participants, 22.1%, 5.9%, 3.9%, and 1.6% had a history of HTN, DM, IHD, or stroke at enrolment, respectively.

Compared with the reference group without any cardiometabolic disease history, the highest age- and sex-adjusted HRs for all-cause and CVD mortality of one condition (stroke) were 1.9 (95% CI, 1.78–2.08) and 3.6 (95% CI, 3.18–3.97), respectively; those for two conditions (stroke and DM) were 3.9 (95% CI, 3.28–4.56) and 5.5 (95% CI, 4.17–7.13), respectively; and those for three conditions (stroke, IHD, and DM) were 4.3 (95% CI, 2.76–6.58) and 10.6 (95% CI, 6.16–18.25), respectively (Table 2 and Table 3).

**Table 2.** Association with past cardiometabolic diseases for the risk of death from all-cause (except S/T codes) in the Asia Cohort Consortium (ACC) from 1984 to 2006

	Cohorts	Death^a^	HR (95% CI)^a^	Death^b^	HR (95% CI)^b^
**Per disease combination**					

None	351,091	41,659	1.00	26,347	1.00

HTN	84,165	16,309	1.17 (1.15–1.19)	538	1.24 (1.21–1.27)
DM	14,140	4,070	1.84 (1.78–1.90)	2,580	2.19 (2.10–2.28)
IHD	7,514	1,995	1.45 (1.39–1.52)	982	1.60 (1.50–1.70)
Stroke	1,763	682	1.92 (1.78–2.08)	388	2.36 (2.12–2.62)

DM, HTN	9,120	3,155	2.10 (2.02–2.17)	1,879	2.62 (2.50–2.75)
IHD, HTN	7,861	2,167	1.45 (1.38–1.51)	980	1.53 (1.43–1.63)
IHD, DM	846	370	2.46 (2.22–2.12)	213	3.37 (2.94–3.86)
Stroke, IHD	155	67	1.81 (1.43–2.30)	28	1.83 (1.22–2.73)
Stroke, HTN	3,245	1,335	2.21 (2.09–2.33)	673	2.61 (2.41–2.81)
Stroke, DM	236	142	3.87 (3.28–4.56)	58	5.80 (4.71–7.14)
					
IHD, DM, HTN	1,590	690	2.60 (2.41–2.80)	361	3.42 (3.09–3.79)
Stroke, IHD, HTN	688	327	2.29 (2.05–2.55)	132	2.45 (2.05–2.91)
Stroke, DM, HTN	745	406	3.47 (3.15–3.83)	243	4.89 (4.30–5.55)
Stroke, IHD, DM	28	20	4.26 (2.76–6.58)	14	7.38 (4.38–12.44)

Stroke, DM, IHD, HTN	345	179	3.01 (2.60–3.49)	94	4.06 (3.31–4.97)

**Per the number of diseases**					

None	351,091	41,659	1.00	26,347	1.00
1 disease	107,582	23,056	1.29 (1.27–1.31)	12,408	1.41 (1.38–1.45)
2 diseases	21,463	7,236	1.89 (1.84–1.94)	3,824	2.27 (2.20–2.36)
3 diseases	3,051	1,443	2.72 (2.58–2.87)	770	3.56 (3.31–3.82)
4 diseases	345	179	3.02 (2.60–3.49)	94	4.09 (3.34–5.01)

DM, diabetes mellitus; HTN, hypertension; IHD, ischemic heart diseases, including myocardial infarction.

S/T codes in International Classification of Diseases 10^th^ edition measured the disability of each injury diagnosis.

^a^Adjusted for age and sex.

^b^Premature death: Age of death <75 years in male and female.

**Table 3.** Association with past cardiometabolic diseases for the risk of death from CVD in the Asia Cohort Consortium (ACC) from 1984 to 2006

	Cohorts	Death^a^	HR (95% CI)^a^	Death^b^	HR (95% CI)^b^
**Per disease combination**					

None	351,091	10,098	1.00	5,739	1.00

HTN	84,165	5,860	1.65 (1.60–1.71)	3,010	2.62 (1.93–2.10)
DM	14,140	1,170	2.18 (2.02–2.28)	723	2.86 (2.64–3.09)
IHD	7,514	851	2.29 (2.13–2.46)	417	3.20 (2.89–3.58)
Stroke	1,763	326	3.55 (3.18–3.97)	168	5.31 (4.55–6.20)

DM, HTN	9,120	1,147	2.91 (2.79–3.15)	592	4.45 (4.11–4.22)
IHD, HTN	7,861	1,052	2.56 (2.40–2.73)	45	3.26 (2.95–3.59)
IHD, DM	846	171	4.23 (3.64–4.92)	108	7.77 (6.82–9.41)
Stroke, IHD	155	35	3.54 (2.54–4.93)	15	5.27 (3.27–8.76)
Stroke, HTN	3,245	731	4.67 (4.32–5.03)	374	6.64 (5.97–7.38)
Stroke, DM	236	54	5.45 (4.17–7.13)	36	10.85 (7.67–14.78)
					
IHD, DM, HTN	1,590	329	4.95 (4.08–5.08)	18	7.43 (6.41–8.62)
Stroke, IHD, HTN	688	204	5.28 (4.59–6.07)	89	7.56 (6.12–9.33)
Stroke, DM, HTN	745	201	6.46 (5.62–7.44)	122	11.18 (9.33–13.39)
Stroke, IHD, DM	28	13	10.60 (6.16–18.25)	9	21.76 (11.32–41.85)

Stroke, DM, IHD, HTN	345	100	6.18 (5.08–7.53)	56	10.98 (8.43–14.30)

**Per the number of diseases**					

None	351,091	10,098	1.00	5,739	1.00
1 disease	107,582	8,207	1.80 (1.74–1.85)	4,318	2.25 (2.16–2.35)
2 diseases	21,463	3,190	3.13 (3.00–3.26)	1,670	4.50 (4.25–4.76)
3 diseases	3,051	747	5.18 (4.80–5.58)	404	8.36 (7.54–9.26)
4 diseases	345	100	6.13 (5.03–7.46)	56	10.87 (8.34–14.15)

CVD, cardiovascular disease; DM, diabetes mellitus; HTN, hypertension; IHD, ischemic heart diseases, including myocardial infarction.

S/T codes in International Classification of Diseases 10^th^ edition measured the disability of each injury diagnosis.

^a^Adjusted for age and sex.

^b^Premature death: Age of death <75 years in male and female.

In the additional analyses adjusted for age, sex, cigarette smoking status, BMI, and cohorts, the pattern of increase in the HRs of cardiometabolic risk factors for all-cause and CVD mortality, including premature death, was broadly similar to those adjusted for age and sex alone (eTable 2).

When accounting for competing risks, the highest adjusted SHRs for CVD mortality and non-CVD mortality showed attenuated patterns compared with the CPH model. For individuals with stroke, the SHRs were 3.45 (95% CI, 3.06–3.90) for CVD mortality and 1.19 (95% CI, 1.06–1.33) for non-CVD mortality. For stroke and DM, the SHRs were 5.08 (95% CI, 4.67–5.52) for CVD mortality and 2.67 (95% CI, 2.10–3.40) for non-CVD mortality. For stroke, IHD, and DM, the SHRs were 11.44 (95% CI, 6.51–20.11) for CVD mortality and 1.27 (95% CI, 0.56–2.92) for non-CVD mortality (eTable 3).

Although the patterns of HRs for premature all-cause and CVD mortality were broadly similar, the risks were more marked for participants with total and sex-specific premature all-cause and CVD mortality (Table 2, Table 3, and eTable 4). Sensitivity analyses excluding participants who died in the first 2 or 5 years of follow-up showed similar patterns. (eTable 5 and eTable 6).

The sex-stratified HRs for all-cause and CVD mortality in men and women were consistent with the overall results (eTable 7 and eTable 8). The HRs of IHD for all-cause and CVD mortality were higher in men than in women, whereas the HRs of other cardiometabolic diseases (HTN, DM, and stroke) for all-cause and CVD mortality were higher in women than in men. Furthermore, the HRs of other cardiovascular multimorbidity for all-cause and CVD mortality were higher in men than in women. The HRs for the combination of stroke, IHD, and DM for premature all-cause and CVD mortality were not estimated in women due to the small number of participants.

In analyses of stroke mortality subtypes (ischemic stroke and hemorrhagic stroke), HTN consistently showed higher HRs than CVD mortality in both types (eTable 9). For ischemic stroke, the HR was 1.97 (95% CI, 1.77–2.18), while for hemorrhagic stroke, the HR was 2.03 (95% CI, 1.88–2.20). Among the two-multimorbidity combinations, stroke and HTN demonstrated the highest HRs for mortality across both ischemic and hemorrhagic strokes. For ischemic stroke mortality, the HR was 8.68 (95% CI, 7.15–10.54), while for hemorrhagic stroke mortality, the HR was 6.80 (95% CI, 5.74–8.05). Overall, cardiovascular multimorbidity involving stroke consistently showed the highest HRs for both ischemic and hemorrhagic stroke mortality (eTable 9).

The risks of individual cardiometabolic diseases and the combination of stroke and DM were consistent across the ACC, ERFC, and UK Biobank, but for other cardiometabolic multimorbidity, HRs were generally lower in the ACC compared with ERFC and UK Biobank (eTable 10). The HRs of all-cause and CVD mortality were similar in the overall patterns, including IHD and MI, for cardiometabolic multimorbidity (eTable 11).

The PAF analysis demonstrated that HTN had the highest PAF for all-cause mortality (4.2%; 95% CI, 3.8–4.6%) and CVD mortality (11.4%; 95% CI, 10.6–12.2%) (Table 4). DM also contributed significantly to the PAF for all-cause mortality (2.4%; 95% CI, 2.3–2.6%) and CVD mortality (3.3%; 95% CI, 2.9–3.6%). Among multimorbidity combinations, HTN with DM had the highest PAF for CVD mortality (3.9%; 95% CI, 3.5–4.2%), followed by stroke with HTN (2.9%; 95% CI, 2.6–3.2%) (Table 4 and eTable 12).

**Table 4.** PAF of cardiometabolic diseases for all-cause and CVD mortality

	Age-standardized prevalence, %	PAF, % (95% CI)^a^	PAF, % (95% CI)^b^
HTN	17.619	4.219 (3.808–4.632)	11.396 (10.624–12.170)
DM	2.881	2.446 (2.277–2.619)	3.261 (2.901–3.645)
IHD	1.757	0.888 (0.775–1.005)	2.350 (2.074–2.642)
Stroke	0.449	0.443 (0.375–0.515)	1.185 (1.006–1.382)

DM, HTN	1.876	2.309 (2.161–2.461)	3.877 (3.531–4.239)
IHD, HTN	1.782	1.041 (0.918–1.169)	3.058 (2.755–3.379)
IHD, DM	0.190	0.311 (0.258–0.371)	0.666 (0.538–0.813)
Stroke, IHD	0.043	0.038 (0.02–0.061)	0.116 (0.069–0.181)
Stroke, HTN	0.737	1.028 (0.927–1.136)	2.903 (2.628–3.202)
Stroke, DM	0.053	0.159 (0.123–0.203)	0.242 (0.168–0.341)

IHD, DM, HTN	0.450	0.847 (0.746–0.956)	1.780 (1.539–2.049)
Stroke, IHD, HTN	0.174	0.267 (0.218–0.322)	0.824 (0.686–0.983)
Stroke, DM, HTN	0.165	0.476 (0.409–0.551)	1.013 (0.848–1.204)
Stroke, IHD, DM	0.007	0.026 (0.013–0.048)	0.073 (0.035–0.141)

Stroke, DM, IHD, HTN	0.085	0.198 (0.156–0.248)	0.483 (0.374–0.62)

CVD, cardiovascular disease; DM, diabetes mellitus; HTN, hypertension; IHD, ischemic heart diseases, including myocardial infarction; PAF, population attributable fraction.

^a^All-cause mortality.

^b^CVD mortality.

## DISCUSSION

This study reports the effects of a combination of hard cardiovascular outcomes (IHD and stroke) and cardiovascular risk factors (HTN and DM) on long-term all-cause and CVD mortality risk. We assessed the additive, sub-additive, multiplicative, synergistic, or antagonistic effects of cardiometabolic multimorbidity on the risk of all-cause and CVD mortality. These effects are provided in eTable 13.^34^^,^^35^

These findings highlight that the combination of stroke and IHD, as well as stroke and DM, showed an additive effect on all-cause mortality (eFigure 3A). For premature all-cause mortality, the combinations of stroke and DM, as well as stroke, IHD, and DM, showed a synergistic effect, implying a higher-than-expected risk based on individual disease risks. Conversely, other combinations showed a sub-additive effect, suggesting a lower-than-expected risk (eFigure 3B).

For the risk of CVD mortality, combinations of IHD and DM; stroke and IHD; and stroke, IHD, and DM showed additive, antagonistic, and synergistic effects, respectively (eFigure 3C). A similar pattern was observed for premature CVD mortality, with an additive effect for stroke and HTN and synergistic effects for the combinations of IHD and DM; stroke and DM; stroke, DM, and HTN; and stroke, IHD, and DM (eFigure 3D). The remaining combinations showed sub-additive effects on premature mortality.

HTN had sub-additive effects on the risk of all-cause and CVD mortality associated with cardiometabolic multimorbidity. In a previous study, the risk of cardiometabolic multimorbidity showed an independent summation of the individual cardiometabolic disease risks (multiplicative effects).^7^ However, the sub-additive effects observed in our study may reflect the complex interplay between hypertension and other cardiometabolic multimorbidity in influencing mortality risk. This can suggest that blood pressure management plays a role in modifying the mortality risk associated with cardiometabolic multimorbidity.^36^^–^^39^ Furthermore, cardiometabolic multimorbidity had sub-additive effects on the risk of all-cause and CVD mortality without considering the HTN effect (eTable 10).^40^^–^^42^

The risk of IHD for all-cause and CVD mortality was lower in Asian cohort studies than in European and North American cohort studies.^7^ As with HTN, the all-cause and CVD mortality risks of cardiometabolic multimorbidity, including IHD, had sub-additive effects, unlike the findings of a previous ERFC study.^7^

Furthermore, we reported that HTN had the highest PAF for both all-cause and CVD mortality, suggesting its dominant contribution to mortality risk. However, compared to other multimorbidity combinations, those involving HTN showed relatively lower PAFs, suggesting that improvements in HTN management may have mitigated some of its mortality risks.

Unlike HTN, DM had a synergistic effect on the risk of CVD mortality associated with cardiometabolic multimorbidity, which was more prominent in premature mortality. Excessive blood-sugar control increases stroke mortality risk^43^; however, randomized controlled trials have not consistently identified the benefit of strict blood-sugar control in reducing stroke mortality risk.^44^ Conversely, HTN management reportedly reduces stroke risk in patients with DM.^44^ International guidelines recommend the routine use of antihypertensives to reduce cardiovascular risk in all patients with DM, including Asians.^45^

Nevertheless, our study primarily focuses on baseline disease status, as data on treatment adherence or changes in therapeutic strategies during the follow-up period were not available. Therefore, we could not adequately account for the potential impact of long-term treatment adherence or adjustments in management strategies over time on the observed mortality risks associated with HTN or DM. These considerations can suggest the importance of further research to better understand the associations between baseline treatment conditions, follow-up adherence, and the observed outcomes of cardiometabolic multimorbidity on mortality. Incorporating detailed treatment histories and adherence data in future analyses could offer valuable insights into how therapeutic interventions shape long-term mortality, particularly in the context of cardiometabolic multimorbidity.

The general patterns of associations remained consistent between the Fine-Gray model and the CPH model. However, compared to the CPH model, the Fine-Gray model showed slightly attenuated HRs for most combinations, reflecting the competing influence of non-CVD mortality. This indicates that the absolute risks of CVD mortality are diminished when non-CVD mortality is considered. In most cases, SHRs for non-CVD mortality were lower or closer to 1.00 compared to the CVD mortality risk. This suggests that cardiovascular multimorbidity predominantly influences CVD mortality rather than non-CVD mortality. However, combinations involving stroke and DM consistently demonstrated strong associations with both CVD and non-CVD mortality, suggesting a notable contribution to overall mortality risk. These findings highlight the potential importance of prevention and management strategies that consider both CVD and non-CVD risks in populations with cardiovascular multimorbidity.

The diagnosis of IHD and MI depends on the patient’s symptoms, electrocardiographic changes, blood biochemistry tests, and imaging.^46^ Since our study depends on self-reported data collected at baseline, distinguishing subtypes of IHD, particularly stable angina and MI, may have been challenging. Stable angina is a chronic and predictable condition within IHD, whereas MI is an acute and irreversible event.^46^ To account for potential variability in definitions, we conducted a sensitivity analysis using MI instead of IHD for a history of cardiometabolic diseases, and the results remained consistent.

Although stroke and IHD affect the long-term mortality risk in survivors more than other diseases. According to our study, stroke or IHD participants with DM or HTN had a significantly higher risk of long-term mortality compared to those without these comorbidities. In particular, the combination of stroke and DM had an additive effect on all-cause mortality and a synergistic effect on premature all-cause mortality. These findings suggest that stroke or IHD survivors with DM or HTN are more likely to have a poorer long-term prognosis than those without DM or HTN and that survivors with comorbidities require more aggressive intervention or closer follow-up.

In this study, women had a higher risk than men for overall mortality from cardiometabolic diseases (except IHD) and a higher risk of cardiometabolic multimorbidity for premature all-cause and CVD mortality. Because sex hormones, such as estrogens and androgens, act as protective factors in premenopausal women, postmenopausal women are at a higher risk of HTN and DM owing to the loss of this protection, which increases CVD risk.^47^ However, women are more likely to be affected by multimorbidity than men because men who live longer tend to be healthier than women of the same age.^48^

Our findings indicate that hemorrhagic stroke is generally associated with higher baseline mortality risks than ischemic stroke, consistent with previous studies.^8^ In Asian countries, stroke, particularly hemorrhagic stroke, is more prevalent compared to European and North American populations. Stroke is the leading cause of cardiovascular mortality in the aging Asian population, underscoring the need for stricter blood pressure control in this group.^8^ Nevertheless, the high risks observed for stroke and multimorbidity combinations including stroke may be partly attributed to the redistribution of a significant proportion of stroke deaths under categories such as “unspecified stroke,” “hypertension,” and various other codes. Ideally, the global burden of stroke should be based on “gold standard” stroke epidemiology data. However, variations in the validity of stroke subtype diagnoses have been highlighted due to the lack of consensus among global experts on the classification of causes of mortality in patients who die post-stroke in clinical trial designs.

Except for the combination of stroke and DM, the risk for other forms of cardiometabolic multimorbidity was generally lower in the ACC compared to the ERFC and UK Biobank. A key factor contributing to this difference is the inclusion of HTN in the definition of multimorbidity in ACC. In Asian populations, the risk associated with HTN as part of multimorbidity appears to be lower compared to its impact in European and North American populations. This may reflect regional differences in HTN management and outcomes.

Our study acknowledges that the individual impacts of HTN, DM, IHD, and stroke on all-cause and CVD mortality are well-established in the previous literature, with prevention and management strategies already implemented in many settings. Nevertheless, our findings suggest that certain combinations, particularly those involving stroke and DM, may be associated with higher risks in the Asian population. In addition, the inclusion of HTN in multimorbidity tends to attenuate the associated risks of mortality compared to other disease combinations. This may reflect the possibility of unique characteristics of HTN in Asian populations, where factors such as responsiveness to blood pressure management could potentially play a role. Therefore, our study can provide additional insights into the cumulative burden of cardiovascular multimorbidity, particularly in Asian populations. This study can offer a contribution to the epidemiological understanding of how specific disease combinations may influence mortality outcomes in this region.

In addition, the previous literature demonstrates variability in how multimorbidity patterns are defined. Prados-Torres et al (2014) systematically reviewed multimorbidity patterns and found no universal agreement among studies on the diseases grouped under this term.^28^ However, they noted recurring clusters of HTN, DM, heart disease, and obesity, consistent with metabolic syndrome. Similarly, Canoy et al (2021) highlighted that cardiometabolic research frequently includes stroke and myocardial infarction, further supporting our rationale for defining cardiometabolic multimorbidity as encompassing these conditions.^29^

This study has several limitations. First, participants included in the ACC are not representative of the overall Asian population, and patients with multimorbidity in the ACC are likely to have milder conditions than the general population, which could have led to an overestimation of the observed associations. Second, the study only considered the association between multimorbidity and mortality and did not examine other outcomes, such as quality of life or disability. Third, residual confounding by unmeasured variables, such as differences in lifestyle or other health behaviors between Asian and European populations, could explain the observed differences in risk. Fourth, the sequence of onset in the participants with cardiometabolic multimorbidity was not determined. The prognostic significance of HTN and DM may vary between individuals with and without pre-existing cardiovascular conditions, potentially influencing the magnitude and direction of mortality risks. Therefore, it is unclear whether there was a causal association in participants with two or more diseases or whether they were due to general risk factors. In addition, the definitions of HTN, DM, IHD, and stroke in this study were based on self-reported data collected at baseline across the ACC. While this approach aligns with the methodology of prior ACC-based studies,^25^^–^^27^ it may introduce potential biases, such as recall bias or misclassification of diseases. Furthermore, the cohort studies included were initiated between 1984 and 2006, which may not fully reflect current advancements in clinical management and healthcare systems. Consequently, the mortality risks observed in this study could be higher than those in more recent cohorts, given the significant improvements in managing cardiometabolic multimorbidity.

Despite these limitations, this study makes an important contribution to CVD research. These findings provide novel insights into the association between multimorbidity and mortality in the understudied Asian populations. We quantified the risks of all-cause and CVD mortality associated with combinations of multiple cardiometabolic diseases in more detail than in previous studies based on a large consortium of multiple cohort studies to increase the sample size and statistical power. Previous studies on the prevalence of multimorbidity included young and middle-aged adults,^49^ whereas a systematic review on the effect of multimorbidity on long- and short-term mortality was limited to older adults.^48^ Risk factors assessed in older adults, especially health-related behaviors, have limitations due to reverse causality.^50^ However, the mean age of our cohort was 54.3 years, and it included middle-aged adults. Furthermore, this study highlights the importance of considering the cultural and geographical differences in CVD management.

This study highlights the importance of addressing complex cardiometabolic diseases, especially in Asian populations, to mitigate long-term mortality risk. Particularly in Asia, unlike in European and North American populations, the combination of stroke and DM is associated with the highest risk of death from all causes and death from cardiovascular disease. Therefore, to extend life expectancy and reduce mortality in Asian populations, tailored prevention strategies through public health interventions that consider the unique epidemiological patterns of Asian populations are needed, with a particular focus on preventing and effectively managing diseases such as stroke and diabetes.

## SUPPLEMENTARY MATERIAL

eTable 1. Equations for the association between cardiometabolic diseases and cardiometabolic multimorbidity on the mortality using the cox proportion hazard regression model

eTable 2. Association with past cardiometabolic diseases for the risk of death from all-cause (except S/T codes) and CVD in the Asia Cohort Consortium (ACC) from 1984 to 2006

eTable 3. Sub-distribution hazard ratio (SHR) and 95% CI for SHR obtained from Fine-Gray competing risk regression models for the association with past cardiometabolic multimorbidity for the risk of death from CVD versus Non-CVD in the Asia Cohort Consortium (ACC) from 1984 to 2006

eTable 4. Association with past cardiometabolic diseases for the risk of premature death from all-cause and CVD in Asia Cohort Consortium (ACC) from 1984 to 2006

eTable 5. Association with past cardiometabolic diseases for the risk of death from all-cause and CVD in Asia Cohort Consortium (ACC): excluding deaths within 2 years of cohort enrollment

eTable 6. Association with past cardiometabolic diseases for the risk of death from all-cause and CVD in Asia Cohort Consortium (ACC): excluding deaths within 5 years of cohort enrollment

eTable 7. Association with past cardiometabolic diseases for the risk of death from all-cause (except S/T codes) among men and women in Asia Cohort Consortium (ACC) from 1984 to 2006

eTable 8. Association with past cardiometabolic diseases for the risk of death from CVD among men and women in Asia Cohort Consortium (ACC) from 1984 to 2006

eTable 9. Association with past cardiometabolic diseases for the risk of death from ischemic stroke and hemorrhagic stroke in the Asia Cohort Consortium (ACC) from 1984 to 2006

eTable 10. Association with past cardiometabolic diseases for the risk of premature death from all-cause and CVD in Asia Cohort Consortium (ACC) from 1984 to 2006 compared with ERFC and UK biobank

eTable 11. Sensitivity analysis for the association with past cardiometabolic diseases for the risk of death from all-cause and CVD according to the definition of IHD/MI in Asia Cohort Consortium (ACC) from 1984 to 2006

eTable 12. Age-standardized prevalence of cardiometabolic diseases based on Eastern Asia population for the year 2000 from World Population Prospects 2022

eTable 13. Description of the types of effects of cardiometabolic multimorbidity on the risk of all-cause and CVD mortality

eFigure 1. Death rates and YLLs per 100K for all-cause mortality based on GBD according to region. (**A**) Deaths in East Asia, (**B**) Deaths in North America, (**C**) Deaths in European, (**D**) YLLs in East Asia, (**E**) YLLs in North America, and (**F**) YLLs in European. GBD, Global Burden of Disease (available from https://vizhub.healthdata.org/gbd-results/); YLL, Years of life lost.

eFigure 2. Death rates and YLLs per 100K for CVD mortality based on GBD according to region. (**A**) Deaths in East Asia, (**B**) Deaths in North America, (**C**) Deaths in European, (**D**) YLLs in East Asia, (**E**) YLLs in North America, and (**F**) YLLs in European. CVD, cardiovascular disease; GBD, Global Burden of Disease (available from https://vizhub.healthdata.org/gbd-results/); YLL, Years of life lost.

eFigure 3. Risk of each cardiometabolic morbidity on all-cause and cardiovascular disease mortality. The cumulative color bars in circular plot are the expected risks of cardiometabolic multimorbidity associated with individually with either disease, whereas the blue dot is the observed risk of cardiometabolic multimorbidity. (**A**) the risk for all-cause mortality, (**B**) the risk for premature all-cause mortality (age of death <70 in men and women), (**C**) the risk for CVD mortality, (**D**) the risk for premature CVD mortality (age of death <70 in men and women). CVD, cardiovascular disease; DM, diabetes mellitus; HTN, hypertension.
