# Supplementary material for: Association Between Cardiometabolic Multimorbidity and 15-year Mortality in the Asia Cohort Consortium
Source: J Epidemiol. 2025 Jul 5;35(7):321–9. doi: 10.2188/jea.JE20240362 (PMC12162181; doi:10.2188/jea.JE20240362)
Supplement: Supplementary file 1 [file je-35-321-s001.pdf]

eTable 1. Equations for the association between cardiometabolic diseases and cardiometabolic multimorbidity on the mortality using the cox proportion hazard regression model

General description of equations
$$h_i(t) = h_0(t)\exp(\beta_1 x_{i1} + \beta_2 x_{i2} + \cdots + \beta_j x_{ij})$$
i: individual
j: cardiometabolic diseases and cardiometabolic multimorbidity
$h_i(t)$: the hazard function for individual i
$h_0(t)$: the baseline hazard function
$x_{i1}, x_{i2}, \dots, x_{ij}$: exposure and covariates
$\beta_1, \beta_2, \dots, \beta_j$: estimated coefficients
j=1: HTN
j=2: DM
j=3: IHD
j=4: Stroke
j=5: DM and HTN
j=6: IHD and HTN
j=7: IHD and DM
j=8: Stroke and IHD
j=9: Stroke and HTN
j=10: Stroke and DM
j=11: IHD, DM, and HTN
j=12: Stroke, IHD, and HTN
j=13: Stroke, DM, and HTN
j=14: Stroke, IHD, and DM
j=15: Stroke, DM, IHD, and HTN
j=16: None (reference group)

DM, diabetes mellitus; HTN, hypertension; IHD, ischemic heart disease.

eTable 2. Association with past cardiometabolic diseases for the risk of death from all-cause (except S/T codes) and CVD in the Asia Cohort Consortium (ACC) from 1984 to 2006

		**All-cause (except S/T codes)**				**CVD**			
	Cohorts	Death	HR (95% CI)[a]	HR (95% CI)[b]	HR (95% CI)[c]	Death	HR (95% CI)[a]	HR (95% CI)[b]	HR (95% CI)[c]
**Per disease combination**									
None	351,091	41,659	1.00	1.00	1.00	10,098	1.00	1.00	1.00
HTN	84,165	16,309	1.20 (1.18–1.22)	1.25 (1.22–1.27)	1.29 (1.26–1.31)	5,860	1.69 (1.64–1.75)	1.73 (1.68–1.79)	1.76 (1.71–1.82)
DM	14,140	4,070	1.84 (1.78–1.90)	1.87 (1.81–1.93)	1.86 (1.80–1.92)	1,170	2.14 (2.02–2.28)	2.17 (2.04–2.31)	2.12 (2.00–2.25)
IHD	7,514	1,995	1.49 (1.43–1.56)	1.51 (1.44–1.57)	1.47 (1.40–1.53)	851	2.36 (2.19–2.53)	2.37 (2.21–2.54)	2.28 (2.12–2.45)
Stroke	1,763	682	1.99 (1.85–2.15)	1.99 (1.84–2.14)	2.08 (1.93–2.24)	326	3.68 (3.29–4.11)	3.67 (3.28–4.10)	3.91 (3.50–4.37)
DM, HTN	9,120	3,155	2.16 (2.08–2.24)	2.26 (2.18–2.34)	2.29 (2.21–2.38)	1,147	3.06 (2.88–3.25)	3.15 (2.96–3.36)	3.13 (2.94–3.33)
IHD, HTN	7,861	2,167	1.52 (1.45–1.59)	1.59 (1.52–1.66)	1.59 (1.52–1.66)	1,052	2.69 (2.52–2.87)	2.77 (2.59–2.95)	2.76 (2.58–2.95)
IHD, DM	846	370	2.54 (2.29–2.82)	2.64 (2.38–2.93)	2.52 (2.28–2.80)	171	4.40 (3.78–5.11)	4.52 (3.88–5.26)	4.21 (3.61–4.90)
Stroke, IHD	155	67	1.89 (1.49–2.4)	1.90 (1.49–2.41)	2.08 (1.64–2.65)	35	3.70 (2.66–5.17)	3.72 (2.67–5.18)	4.12 (2.95–5.74)
Stroke, HTN	3,245	1,335	2.33 (2.21–2.47)	2.41 (2.28–2.55)	2.67 (2.52–2.82)	731	4.96 (4.59–5.35)	5.06 (4.69–5.46)	5.60 (5.19–6.05)
Stroke, DM	236	142	3.84 (3.26–4.53)	4.00 (3.39–4.72)	4.24 (3.59–5.00)	54	5.42 (4.15–7.08)	5.57 (4.26–7.29)	5.90 (4.51–7.71
IHD, DM, HTN	1,590	690	2.74 (2.54–2.96)	2.90 (2.69–3.13)	2.83 (2.62–3.06)	329	4.85 (4.34–5.41)	5.03 (4.50–5.61)	4.82 (4.31–5.39)
Stroke, IHD, HTN	688	327	2.44 (2.19–2.72)	2.54 (2.27–2.83)	2.91 (2.61–3.24)	204	5.64 (4.90–6.48)	5.78 (5.03–6.65)	6.63 (5.76–7.63)
Stroke, DM, HTN	745	406	3.70 (3.36–4.09)	3.90 (3.53–4.30)	4.16 (3.77–4.59)	201	6.95 (6.04–8.00)	7.20 (6.26–8.28)	7.55 (6.56–8.69)
Stroke, IHD, DM	28	20	4.94 (3.19–7.63)	5.04 (3.26–7.78)	5.54 (3.58–8.56)	13	12.16 (7.06–20.93)	12.22 (7.10–21.04)	13.39 (7.77–23.05)
Stroke, DM, IHD, HTN	345	179	3.18 (2.74–3.68)	3.33 (2.88–3.86)	3.73 (3.22–4.32)	100	6.50 (5.34–7.92)	6.70 (5.50–8.17)	7.42 (6.08–9.04)
**Per the number of diseases**									
None	351,091	41,659	1.00	1.00	1.00	10,098	1.00	1.00	1.00
1 disease	107,582	23,056	1.32 (1.30–1.34)	1.36 (1.34–1.39)	1.40 (1.38–1.42)	8,207	1.84 (1.78–1.89)	1.88 (1.82–1.94)	1.90 (1.84–1.96)
2 diseases	21,463	7,236	1.97 (1.92–2.02)	2.06 (2.01–2.11)	2.11 (2.06–2.17)	3,190	3.26 (3.13–3.4)	3.37 (3.24–3.52)	3.41 (3.27–3.55)
3 diseases	3,051	1,443	2.89 (2.74–3.05)	3.05 (2.89–3.22)	3.18 (3.01–3.35)	747	5.53 (5.12–5.96)	5.74 (5.32–6.19)	5.88 (5.45–6.35)
4 diseases	345	179	3.18 (2.75–3.68)	3.35 (2.90–3.88)	3.75 (3.24–4.34)	100	6.44 (5.28–7.84)	6.67 (5.48–8.13)	7.36 (6.04–8.97)

CVD, cardiovascular disease; DM, diabetes mellitus; HTN, hypertension; IHD, ischemic heart diseases, including myocardial infarction.
S/T codes in International Classification of Diseases 10th edition measured the disability of each injury diagnosis
[a] Adjusted for age, sex, and cigarette smoking
[b] Adjusted for age, sex, cigarette smoking, and body mass index
[c] Adjusted for age, sex, cigarette smoking, body mass index, and cohorts

eTable 3. Sub-distribution hazard ratio (SHR) and 95% CI for SHR obtained from Fine-Gray competing risk regression models for the association with past cardiometabolic multimorbidity for the risk of death from CVD versus Non-CVD in the Asia Cohort Consortium (ACC) from 1984 to 2006

	**CVD**	**Non-CVD**
	SHR (95% CI)[a]	SHR (95% CI)[a]
**Per disease combination**		
None	1.00	1.00
HTN	1.76 (1.70–1.82)	1.08 (1.06–1.11)
DM	1.96 (1.84–2.09)	1.67 (1.61–1.74)
IHD	2.23 (2.07–2.41)	1.07 (1.01–1.14)
Stroke	3.45 (3.06–3.90)	
		1.19 (1.06–1.33)
DM, HTN	2.81 (2.63–3.00)	1.78 (1.70–1.87)
IHD, HTN	2.75 (2.57–2.94)	1.02 (0.96–1.09)
IHD, DM	3.73 (3.16–4.41)	1.53 (1.32–1.78)
Stroke, IHD	3.59 (2.45–5.26)	1.15 (0.80–1.66)
Stroke, HTN	4.24 (3.18–5.66)	1.24 (1.13–1.35)
Stroke, DM	5.08 (4.67–5.52)	
		2.67 (2.10–3.40)
IHD, DM, HTN	4.16 (3.69–4.69)	1.65 (1.48–1.85)
Stroke, IHD, HTN	6.17 (5.31–7.17)	1.08 (0.89–1.30)
Stroke, DM, HTN	6.07 (5.21–7.08)	2.00 (1.72–2.32)
Stroke, IHD, DM	11.44 (6.51–20.11)	1.27 (0.56–2.92)
Stroke, DM, IHD, HTN	6.20 (4.95–7.78)	1.60 (1.26–2.02)

CVD, cardiovascular disease; DM, diabetes mellitus; HTN, hypertension; IHD, ischemic heart diseases, including myocardial infarction; SHR, sub-distribution hazard ratio.

[a] Adjusted for age, sex, cigarette smoking, body mass index, and cohorts

eTable 4. Association with past cardiometabolic diseases for the risk of premature death from all-cause and CVD in Asia Cohort Consortium (ACC) from 1984 to 2006

	All-cause (except S/T codes)		CVD	
	Death[a]	HR (95% CI)[b]	Death[a]	HR (95% CI)[b]
Per disease combination				
None	26,347	1.00	5,739	1.00
HTN	8,538	1.24 (1.21–1.27)	3,010	2.09 (2.00–2.19)
DM	2,580	2.19 (2.10–2.28)	723	2.86 (2.65–3.10)
IHD	942	1.60 (1.50–1.70)	417	3.30 (2.98–3.65)
Stroke	348	2.36 (2.12–2.62)	168	5.46 (4.68–6.37)
DM, HTN	1,879	2.62 (2.50–2.75)	692	4.66 (4.30–5.05)
IHD, HTN	940	1.53 (1.43–1.63)	445	3.47 (3.15–3.83)
IHD, DM	213	3.37 (2.94–3.86)	108	8.12 (6.71–9.84)
Stroke, IHD	24	1.83 (1.22–2.73)	15	5.45 (3.28–9.05)
Stroke, HTN	679	2.61 (2.41–2.81)	374	7.13 (6.41–7.94)
Stroke, DM	89	5.80 (4.71–7.14)	36	10.79 (7.77–14.99)
IHD, DM, HTN	381	3.42 (3.09–3.79)	184	8.03 (6.92–9.32)
Stroke, IHD, HTN	132	2.45 (2.06–2.91)	89	8.18 (6.63–10.11)
Stroke, DM, HTN	243	4.89 (4.30–5.55)	122	12.25 (10.22–14.68)
Stroke, IHD, DM	14	7.38 (4.38–12.44)	9	24.94 (12.97–47.96)
Stroke, DM, IHD, HTN	94	4.06 (3.31–4.97)	56	11.73 (9.01–15.28)
Per the number of diseases				
None	26,347	1.00	5,739	1.00
1 disease	12,408	1.48 (1.45–1.52)	4,318	2.33 (2.24–2.43)
2 diseases	3,824	2.45 (2.37–2.54)	1,670	4.79 (4.52–5.07)
3 diseases	770	3.91 (3.63–4.20)	404	9.12 (8.22–10.12)
4 diseases	94	4.49 (3.67–5.50)	56	11.69 (8.97–15.22)

CVD, cardiovascular disease; DM, diabetes mellitus; HTN, hypertension; IHD, ischemic heart diseases, including myocardial infarction.
S/T codes in International Classification of Diseases 10th edition measured the disability of each injury diagnosis
[a] Premature death: Age of death <75 years in men and women
[b] Adjusted for age, sex, cigarette smoking, and body mass index

eTable 5. Association with past cardiometabolic diseases for the risk of death from all-cause and CVD in Asia Cohort Consortium (ACC): excluding deaths within 2 years of cohort enrollment

	**All-cause (except S/T codes)**				**CVD**			
	Death[a]	HR (95% CI)[a]	Death[b]	HR (95% CI)[b]	Death[a]	HR (95% CI)[a]	Death[b]	HR (95% CI)[b]
None	38,681	1.00	23,816	1.00	9,335	1.00	5,169	1.00
HTN	15,123	1.25 (1.23–1.28)	7,610	1.32 (1.29–1.36)	5,311	1.71 (1.66–1.77)	2,595	2.06 (1.96–2.17)
DM	3,708	1.86 (1.79–1.92)	2,265	2.21 (2.11–2.30)	1,065	2.17 (2.03–2.31)	636	2.88 (2.65–3.13)
IHD	1,761	1.47 (1.41–1.55)	776	1.60 (1.49–1.72)	732	2.27 (2.11–2.45)	328	3.07 (2.74–3.44)
Stroke	574	1.90 (1.75–2.06)	274	2.31 (2.05–2.61)	262	3.39 (2.99–3.83)	126	5.01 (4.19–5.98)
DM, HTN	2,846	2.24 (2.16–2.33)	1,611	2.78 (2.64–2.92)	1,008	3.07 (2.88–3.28)	575	4.55 (4.16–4.97)
IHD, HTN	1,954	1.59 (1.51–1.66)	791	1.64 (1.53–1.76)	923	2.71 (2.52–2.9)	359	3.34 (2.99–3.73)
IHD, DM	307	2.46 (2.20–2.16)	160	3.20 (2.74–3.75)	132	3.95 (3.33–4.69)	77	7.05 (5.63–8.84)
Stroke, IHD	59	1.93 (1.49–2.49)	17	1.67 (1.04–2.69)	29	3.59 (2.49–5.17)	9	4.16 (2.16–8.01)
Stroke, HTN	1,186	2.43 (2.30–2.58)	570	2.87 (2.64–3.12)	642	5.11 (4.71–5.54)	312	7.34 (6.53–8.25)
Stroke, DM	116	3.80 (3.17–4.56)	65	5.49 (4.30–7.01)	43	5.20 (3.85–7.02)	25	9.62 (6.49–14.26)
IHD, DM, HTN	596	2.82 (2.60–3.06)	299	3.53 (3.15–3.96)	278	4.82 (4.27–5.43)	139	7.43 (6.27–8.82)
Stroke, IHD, HTN	295	2.64 (2.35–2.96)	113	2.87 (2.38–3.45)	185	6.10 (5.27–7.07)	75	8.78 (6.98–11.05)
Stroke, DM, HTN	342	3.81 (3.42–4.24)	188	5.20 (4.50–6.01)	163	6.85 (5.86–8.00)	90	11.52 (9.34–14.21)
Stroke, IHD, DM	13	3.78 (2.19–6.51)	8	6.28 (3.14–12.54)	8	9.03 (4.54–17.96)	5	18.07 (7.52–43.44)
Stroke, DM, IHD, HTN	152	3.28 (2.79–3.84)	72	4.26 (3.38–5.37)	86	6.74 (5.45–8.34)	43	11.48 (8.49–15.52)
**Per the number of diseases**								
None	38,681	1.00	23,816	1.00	9,335	1.00	5,169	1.00
1 disease	21,166	1.36 (1.34–1.39)	10,925	1.49 (1.45–1.52)	7,370	1.85 (1.79–1.91)	3,685	2.29 (2.19–2.39)
2 diseases	6,468	2.05 (1.99–2.10)	3,214	2.43 (2.33–2.52)	2,777	3.29 (3.14–3.43)	1,357	4.64 (4.36–4.95)
3 diseases	1,246	3.01 (2.84–3.18)	608	3.79 (3.49–4.11)	634	5.61 (5.17–6.09)	309	8.73 (7.76–9.82)
4 diseases	152	3.30 (2.81–3.87)	72	4.30 (3.41–5.42)	86	6.72 (5.43–8.31)	43	11.44 (8.47–15.47)

DM, diabetes mellitus; HTN, hypertension; IHD, ischemic heart diseases, including myocardial infarction.

S/T codes in International Classification of Diseases 10th edition measured the disability of each injury diagnosis

[a] Adjusted for age, sex, cigarette smoking, and body mass index

[b] Premature death: Age of death <75 years in men and women

eTable 6. Association with past cardiometabolic diseases for the risk of death from all-cause and CVD in Asia Cohort Consortium (ACC): excluding deaths within 5 years of cohort enrollment

	**All-cause (except S/T codes)**				**CVD**			
	Death[a]	HR (95% CI)[a]	Death[b]	HR (95% CI)[b]	Death[a]	HR (95% CI)[a]	Death[b]	HR (95% CI)[b]
None	32,841	1.00	19,088	1.00	7,953	1.00	4,156	1.00
HTN	12,715	1.26 (1.24–1.29)	5,900	1.36 (1.32–1.40)	4,366	1.68 (1.62–1.75)	1,957	2.05 (1.94–2.16)
DM	3,050	1.84 (1.77–1.91)	1,743	2.23 (2.12–2.34)	865	2.12 (1.98–2.27)	485	2.88 (2.62–3.17)
IHD	1,402	1.47 (1.39–1.55)	550	1.59 (1.49–1.73)	562	2.15 (1.98–2.35)	224	2.90 (2.53–3.32)
Stroke	418	1.80 (1.63–1.98)	176	2.20 (1.89–2.55)	191	3.19 (2.76–3.69)	85	4.94 (3.98–6.13)
DM, HTN	2,272	2.22 (2.13–2.32)	1,160	2.77 (2.61–2.95)	796	3.00 (2.79–3.23)	414	4.52 (4.07–5.01)
IHD, HTN	1,595	1.64 (1.56–1.72)	579	1.74 (1.60–1.89)	737	2.68 (2.48–2.90)	248	3.27 (2.87–3.73)
IHD, DM	227	2.34 (2.05–2.66)	99	2.92 (2.39–3.55)	88	3.33 (2.70–4.11)	41	5.44 (4.00–7.41)
Stroke, IHD	43	1.93 (1.43–2.61)	10	1.61 (0.86–2.99)	23	3.86 (2.56–5.82)	5	3.70 (1.54–8.90)
Stroke, HTN	882	2.42 (2.26–2.59)	368	2.84 (2.56–3.15)	464.	4.89 (4.45–5.38)	198	7.06 (6.10–8.16)
Stroke, DM	80	3.64 (2.93–4.54)	42	5.86 (4.32–7.93)	33	5.43 (3.86–7.65)	17	10.60 (6.58–17.09)
IHD, DM, HTN	444	2.76 (2.52–3.04)	180	3.23 (2.79–3.75)	204	4.56 (3.96–5.24)	77	6.15 (4.90–7.72)
Stroke, IHD, HTN	230	2.91 (2.55–3.31)	74	3.15 (2.50–3.96)	141	6.43 (5.44–7.61)	48	9.22 (6.92–12.28)
Stroke, DM, HTN	249	3.89 (3.44–4.41)	114	5.15 (4.29–6.20)	114	6.64 (5.51–7.99)	51	10.53 (7.97–13.90)
Stroke, IHD, DM	12	4.89 (2.78–8.62)	7	9.19 (4.38–19.28)	7	10.78 (5.14–22.63)	4	23.84 (8.96–63.40)
Stroke, DM, IHD, HTN	105	3.15 (2.60–3.82)	37	3.56 (2.58–4.92)	54	5.80 (4.43–7.58)	17	7.22 (4.48–11.64)
**Per the number of diseases**								
None	32,841	1.00	19,088	1.00	7,953	1.00	4,156	1.00
1 disease	17,585	1.37 (1.34–1.39)	8,369	1.51 (1.47–1.56)	5,984	1.80 (1.74–1.86)	2,751	2.26 (2.15–2.38)
2 diseases	5,099	2.05 (1.99–2.11)	2,258	2.45 (2.34–2.56)	2,141	3.18 (3.03–3.34)	923	4.49 (4.16–4.84)
3 diseases	935	3.07 (2.87–3.28)	375	3.70 (3.34–4.11)	466	5.51 (5.01–6.06)	180	7.91 (6.80–9.22)
4 diseases	105	3.16 (2.61–3.83)	37	3.59 (2.60–4.96)	54	5.80 (4.44–7.59)	17	7.21 (4.47–11.62)

DM, diabetes mellitus; HTN, hypertension; IHD, ischemic heart diseases, including myocardial infarction.
S/T codes in International Classification of Diseases 10th edition measured the disability of each injury diagnosis
[a] Adjusted for age, sex, cigarette smoking, and body mass index
[b] Premature death: Age of death <75 years in men and women

eTable 7. Association with past cardiometabolic diseases for the risk of death from all-cause (except S/T codes) among men and women in Asia Cohort Consortium (ACC) from 1984 to 2006

	**Men**				**Women**			
Total database	Death[a]	HR (95% CI)[a]	Death[b]	HR (95% CI)[b]	Death[a]	HR (95% CI)[a]	Death[b]	HR (95% CI)[b]
**Per disease combination**								
None	24,472	1.00	16,207	1.00	17,187	1.00	10,140	1.00
HTN	8,937	1.23 (1.20–1.26)	4,994	1.28 (1.24–1.32)[c]	7,312	1.27 (1.23–1.30)	3,544	1.37 (1.32–1.42)[c]
DM	2,496	1.71 (1.64–1.78)[c]	1,649	1.96 (1.86–2.06)	1,574	2.15 (2.04–2.26)[c]	931	2.80 (2.61–2.99)
IHD	1,133	1.57 (1.47–1.66)[c]	590	1.78 (1.64–1.94)	862	1.41 (1.32–1.52)[c]	352	1.46 (1.31–1.63)
Stroke	482	1.83 (1.67–2.00)[c]	262	2.26 (2.00–2.55)	200	2.45 (2.13–2.82)[c]	86	2.88 (2.33–3.56)
DM, HTN	1,677	2.00 (1.90–2.10)[c]	1,061	2.42 (2.27–2.58)[c]	1,478	2.66 (2.52–2.80)[c]	818	3.50 (3.25–3.76)[c]
IHD, HTN	1,078	1.61 (1.51–1.71)	495	1.68 (1.54–1.84)	1,089	1.55 (1.46–1.65)	445	1.62 (1.47–1.78)
IHD, DM	218	2.63 (2.30–3.01)	137	3.57 (3.02–4.23)	152	2.61 (2.23–3.07)	76	3.45 (2.75–4.33)
Stroke, IHD	44	1.59 (1.18–2.13)[c]	16	1.55 (0.95–2.54)	23	3.09 (2.05–4.65)[c]	8	2.93 (1.46–5.86)
Stroke, HTN	892	2.25 (2.11–2.41)[c]	480	2.64 (2.41–2.89)[c]	443	2.87 (2.61–3.16)[c]	199	3.35 (2.91–3.86)[c]
Stroke, DM	101	4.60 (3.79–5.60)[c]	65	6.30 (4.93–8.04)	41	3.09 (2.27–4.20)[c]	24	5.35 (3.58–7.99)
IHD, DM, HTN	320	2.57 (2.30–2.87)[c]	178	3.15 (2.72–3.66)[c]	370	3.27 (2.95–3.63)[c]	203	4.46 (3.88–5.14)[c]
Stroke, IHD, HTN	204	2.31 (2.01–2.66)[c]	84	2.33 (1.88–2.89)[c]	123	3.08 (2.58–3.68)[c]	48	3.57 (2.69–4.74)[c]
Stroke, DM, HTN	247	3.64 (3.21–4.12)[c]	149	4.75 (4.04–5.59)[c]	159	4.53 (3.87–5.29)	94	6.97 (5.69–8.55)[c]
Stroke, IHD, DM	17	5.19 (3.23–8.35)	12	8.76 (4.97–15.42)	3	4.16 (1.34–12.91)	2	7.13 (1.78–28.54)
Stroke, DM, IHD, HTN	103	3.02 (2.49–3.67)	56	3.94 (3.03–5.13)	76	3.96 (3.16–4.96)	38	5.36 (3.89–7.37)
**Per the number of diseases**								
None	24,472	1.00	16,207	1.00	17,187	1.00	10,140	1.00
1 disease	13,048	1.35 (1.32–1.38)	7,495	1.44 (1.40–1.49)	10,008	1.38 (1.35–1.42)	4,913	1.54 (1.49–1.60)
2 diseases	4,010	1.97 (1.91–2.04)	2,254	2.32 (2.22–2.43)	3,226	2.17 (2.09–2.26)	1,570	2.64 (2.50–2.79)
3 diseases	788	2.78 (2.59–2.99)	423	3.39 (3.08–3.74)	655	3.48 (3.22–3.77)	347	4.80 (4.30–5.35)
4 diseases	103	3.05 (2.51–3.70)	56	3.97 (3.05–5.16)	76	3.97 (3.17–4.98)	38	5.37 (3.91–7.39)

CVD, cardiovascular disease; DM, diabetes mellitus; HTN, hypertension; IHD, ischemic heart diseases, including myocardial infarction.
S/T codes in International Classification of Diseases 10th edition measured the disability of each injury diagnosis
[a] Adjusted for age, sex, cigarette smoking, and body mass index
[b] Premature death: Age of death <75 years in men and women
[c] P-values for the difference of two HRs from men and women <0.05

eTable 8. Association with past cardiometabolic diseases for the risk of death from CVD among men and women in Asia Cohort Consortium (ACC) from 1984 to 2006

	**Men**				**Women**			
Total database	Death[a]	HR (95% CI)[a]	Death[b]	HR (95% CI)[b]	Death[a]	HR (95% CI)[a]	Death[b]	HR (95% CI)[b]
**Per disease combination**								
None	5,391	1.00	3,374	1.00	4,707	1.00	2,365	1.00
HTN	2,984	1.78 (1.70–1.87)	1,722	2.10 (1.98–2.23)	2,876	1.68 (1.60–1.76)	1,288	2.08 (1.94–2.24)
DM	642	1.96 (1.81–2.13)[c]	437	2.51 (2.27–2.77)[c]	528	2.46 (2.25–2.69)[c]	286	3.57 (3.16–4.04)[c]
IHD	477	2.80 (2.54–3.07)[c]	280	4.12 (3.64–4.66)[c]	374	1.96 (1.76–2.18)[c]	137	2.33 (1.96–2.77)[c]
Stroke	207	3.36 (2.92–3.86)[c]	109	4.62 (3.81–5.60)[c]	119	4.59 (3.83–5.51)[c]	59	8.04 (6.20–10.42)[c]
DM, HTN	512	2.91 (2.66–3.17)[c]	377	4.09 (3.67–4.55)[c]	575	3.45 (3.16–3.77)[c]	215	5.58 (4.94–6.29)[c]
IHD, HTN	508	3.11 (2.84–3.42)[c]	246	3.99 (3.49–4.56)	544	2.49 (2.27–2.72)[c]	199	2.98 (2.57–3.45)
IHD, DM	104	5.28 (4.35–6.42)[c]	78	9.87 (7.87–12.37)	67	3.69 (2.90–4.70)[c]	30	5.49 (3.82–7.88)
Stroke, IHD	22	3.31 (2.18–5.03)	11	5.38 (2.97–9.74)	13	5.61 (3.26–9.68)	4	5.84 (2.19–15.58)
Stroke, HTN	470	4.94 (4.49–5.44)	254	6.71 (5.89–7.65)	261	5.54 (4.89–6.28)	120	8.39 (6.97–10.11)
Stroke, DM	34	6.32 (4.51–8.86)	23	10.60 (7.03–16.00)	20	4.65 (2.99–7.21)	13	11.23 (6.50–19.41)
IHD, DM, HTN	151	4.95 (4.20–5.82)	88	7.40 (5.98–9.17)	178	5.13 (4.41–5.97)	96	8.68 (7.06–10.68)
Stroke, IHD, HTN	122	5.57 (4.65–6.68)	54	7.19 (5.48–9.43)	82	6.33 (5.08–7.89)	35	10.55 (7.54–14.77)
Stroke, DM, HTN	114	6.88 (5.71–8.30)	72	10.91 (8.62–13.82)	87	7.95 (6.42–9.84)	50	15.26 (11.51–20.24)
Stroke, IHD, DM	12	14.63 (8.32–25.73)	9	30.95 (16.08–59.56)	1	4.58 (0.65–32.56)	0	–
Stroke, DM, IHD, HTN	54	6.27 (4.79–8.20)	32	10.52 (7.42–14.93)	46	7.49 (5.60–10.02)	24	13.59 (9.07–20.34)
**Per the number of diseases**								
None	5,391	1.00	3,374	1.00	4,707	1.00	2,365	1.00
1 disease	4,310	1.93 (1.85–2.01)	2,548	2.34 (2.22–2.47)	3,897	1.82 (1.74–1.90)	1,770	2.32 (2.18–2.48)
2 diseases	1,710	3.49 (3.30–3.69)	989	4.81 (4.46–5.18)	1,480	3.25 (3.06–3.45)	681	4.72 (4.31–5.16)
3 diseases	399	5.66 (5.10–6.28)	223	8.37 (7.28–9.62)	348	5.93 (5.31–6.63)	181	10.25 (8.77–11.97)
4 diseases	54	6.22 (4.75–8.14)	32	10.38 (7.31–14.73)	46	7.50 (5.60–10.02)	24	13.59 (9.08–20.35)

CVD, cardiovascular disease; DM, diabetes mellitus; HTN, hypertension; IHD, ischemic heart diseases, including myocardial infarction.
S/T codes in International Classification of Diseases 10th edition measured the disability of each injury diagnosis
[a] Adjusted for age, sex, cigarette smoking, and body mass index
[b] Premature death: Age of death <75 years in men and women
[c] P-values for the difference of two HRs from men and women <0.05

eTable 9. Association with past cardiometabolic diseases for the risk of death from ischemic stroke and hemorrhagic stroke in the Asia Cohort Consortium (ACC) from 1984 to 2006

	**Ischemic stroke (I63)**		**Hemorrhagic stroke (I60-I62)**	
	Death	HR (95% CI)[a]	Death	HR (95% CI)[a]
**Per disease combination**				
None	897	1.00	1,821	1.00
HTN	636	1.97 (1.77–2.18)[b]	1,123	2.03 (1.88–2.20)[b]
DM	112	2.26 (1.86–2.76)[b]	132	1.46 (1.22–1.75)
IHD	48	1.39 (1.04–1.86)	80	1.38 (1.10–1.72)
Stroke	50	5.98 (4.48–7.97)[b]	79	5.75 (4.58–7.22)[b]
DM, HTN	136	3.85 (3.21–4.63)[b]	147	2.54 (2.14–3.01)
IHD, HTN	95	2.51 (2.02–3.11)[b]	152	2.56 (2.16–3.03)[b]
IHD, DM	10	2.69 (1.44–5.02)	9	1.51 (0.78–2.91)
Stroke, IHD	6	6.67 (2.98–14.90)[b]	6	4.28 (1.92–9.56)[b]
Stroke, HTN	121	8.68 (7.15–10.54)[b]	152	6.80 (5.74–8.05)[b]
Stroke, DM	7	7.30 (3.47–15.38)	8	5.26 (2.63–10.55)
IHD, DM, HTN	30	4.58 (3.17–6.61)[b]	34	3.35 (2.38–4.71)
Stroke, IHD, HTN	27	7.68 (5.22–11.30)[b]	37	7.05 (5.08–9.79)[b]
Stroke, DM, HTN	28	10.06 (6.89–14.69)[b]	35	8.14 (5.81–11.40)[b]
Stroke, IHD, DM	3	29.36 (9.44–91.35)[b]	0	–
Stroke, DM, IHD, HTN	12	7.88 (4.45–13.97) [b]	13	5.75 (3.33–9.94)[b]
**Per the number of diseases**				
None	897	1.00	1,821	1.00
1 disease	846	2.04 (1.85–2.24)	1,414	1.97 (1.84–2.12)
2 diseases	375	4.04 (3.57–4.58)	474	3.17 (2.85–3.52)
3 diseases	88	6.76 (5.41–8.46)	106	5.28 (4.32–6.44)
4 diseases	12	7.83 (4.42–13.87)	13	5.69 (3.29–9.83)

DM, diabetes mellitus; HTN, hypertension; IHD, ischemic heart diseases, including myocardial infarction.
[a] Adjusted for age, sex, cigarette smoking, and body mass index
[b] p-heterogeneity between the HR for all-cause mortality and the HR for CVD mortality <0.05

eTable 10. Association with past cardiometabolic diseases for the risk of premature death from all-cause and CVD in Asia Cohort Consortium (ACC) from 1984 to 2006 compared with ERFC and UK biobank

	**All-cause (except S/T codes)**				**CVD**		
	**ACC**		**ERFC**	**UK biobank**	**ACC**		**ERFC**
Total database	Death	HR (95% CI)[a]	HR (95% CI)	HR (95% CI)	Death	HR (95% CI)[a]	HR (95% CI)
**Per disease combination**							
None	97,968	1.00	1.00	1.00	15,958	1.00	1.00
DM	7,225	1.90 (1.85–1.95)	1.9 (1.8–2.0)	1.6 (1.5–1.8)	2,317	2.13 (2.04–2.23)	2.4 (2.2–2.6)
MI	4,162	1.44 (1.40–1.49)	2.0 (1.9–2.2)	2.1 (1.9–2.3)	1,903	2.10 (2.00–2.21)	3.3 (3.0–3.6)
Stroke	2,017	2.10 (2.00–2.19)	2.1 (2.0–2.2)	2.1 (1.8–2.4)	1,057	3.71 (3.49–3.96)	3.0 (2.8–3.3)
DM, MI	1,060	2.60 (2.45–2.77)	3.7 (3.3–4.1)	4.3 (3.7–5.0)	500	3.92 (3.59–4.29)	6.5 (5.7–7.5)
Stroke, MI	394	2.22 (2.01–2.45)	3.5 (3.1–4.0)	3.6 (2.7–4.7)	239	4.31 (3.79–4.91)	6.9 (5.9–8.0)
Stroke, DM	548	3.65 (3.35–3.97)	3.8 (3.5–4.2)	3.9 (3.7–5.0)	255	5.50 (4.86–6.23)	6.2 (5.4–7.1)
Stroke, MI, DM	199	3.19 (2.77–3.66)	6.9 (5.7–8.3)	5.8 (3.9–8.5)	113	5.68 (4.72–6.84)	11.8 (9.6–14.6)

CVD, cardiovascular disease; DM, diabetes mellitus; ERFC, The Emerging Risk Factors Collaboration; HTN, hypertension; IHD, ischemic heart diseases, including myocardial infarction.
S/T codes in International Classification of Diseases 10th edition measured the disability of each injury diagnosis
[a] Adjusted for age, sex, cigarette smoking, and body mass index

eTable 11. Sensitivity analysis for the association with past cardiometabolic diseases for the risk of death from all-cause and CVD according to the definition of IHD/MI in Asia Cohort Consortium (ACC) from 1984 to 2006

	**All-cause (except S/T codes)**		**CVD**			**All-cause (except S/T codes)**		**CVD**	
Total database	Death	HR (95% CI)[a]	Death	HR (95% CI)[a]	Total database	Death	HR (95% CI)[a]	Death	HR (95% CI)[a]
**Per disease combination**					**Per disease combination**				
None	41,659	1.00	10,098	1.00	None	41,645	1.00	10,090	1.00
HTN	16,309	1.25 (1.22–1.27)	5,860	1.73 (1.68–1.79)	HTN	16,288	1.24 (1.22–1.27)	5,847	1.72 (1.67–1.78)
DM	4,070	1.87 (1.81–1.93)	1,170	2.17 (2.04–2.31)	DM	4,068	1.87 (1.81–1.93)	1,170	2.17 (2.04–2.30)
IHD	1,995	1.51 (1.44–1.57)	851	2.37 (2.21–2.54)	MI	794	1.39 (1.30–1.49)	329	2.20 (1.97–2.46)
Stroke	682	1.99 (1.84–2.14)	326	3.67 (3.28–4.10)	Stroke	681	1.98 (1.84–2.14)	325	3.66 (3.27–4.09)
DM, HTN	3,155	2.26 (2.18–2.34)	1,147	3.15 (2.96–3.36)	DM, HTN	3,147	2.25 (2.17–2.33)	1,141	3.12 (2.93–3.32)
IHD, HTN	2,167	1.59 (1.52–1.66)	1,052	2.77 (2.59–2.95)	MI, HTN	682	1.65 (1.53–1.79)	319	2.90 (2.59–3.24)
IHD, DM	370	2.64 (2.38–2.93)	171	4.52 (3.88–5.26)	MI, DM	122	2.18 (1.82–2.6)	50	3.51 (2.66–4.63)
Stroke, IHD	67	1.90 (1.49–2.41)	35	3.72 (2.67–5.18)	Stroke, MI	30	1.86 (1.30–2.66)	15	3.59 (2.16–5.96)
Stroke, HTN	1,335	2.41 (2.28–2.55)	731	5.06 (4.69–5.46)	Stroke, HTN	1,330	2.40 (2.27–2.53)	728	5.03 (4.66–5.43)
Stroke, DM	142	4.00 (3.39–4.72)	54	5.57 (4.26–7.29)	Stroke, DM	141	3.99 (3.38–4.71)	53	5.49 (4.19–7.19)
IHD, DM, HTN	690	2.90 (2.69–3.13)	329	5.03 (4.50–5.61)	MI, DM, HTN	165	2.41 (2.07–2.81)	64	3.52 (2.75–4.5)
Stroke, IHD, HTN	327	2.54 (2.27–2.83)	204	5.78 (5.03–6.65)	Stroke, MI, HTN	105	2.48 (2.05–3.01)	64	5.56 (4.35–7.12)
Stroke, DM, HTN	406	3.90 (3.53–4.30)	201	7.20 (6.26–8.28)	Stroke, DM, HTN	405	3.90 (3.53–4.30)	200	7.17 (6.23–8.25)
Stroke, IHD, DM	20	5.04 (3.26–7.78)	13	12.22 (7.10–21.04)	Stroke, MI, DM	8	4.44 (2.23–8.84)	5	10.81 (4.51–25.92)
Stroke, DM, IHD, HTN	179	3.33 (2.88–3.86)	100	6.70 (5.50–8.17)	Stroke, DM, MI, HTN	56	2.28 (1.75–2.96)	32	4.70 (3.32–6.66)
**Per the number of diseases**					**Per the number of diseases**				
None	41,659	1.00	10,098	1.00	None	41,645	1.00	10,090	1.00
1 disease	23,056	1.36 (1.34–1.39)	8,207	1.88 (1.82–1.94)	1 disease	21,831	1.35 (1.33–1.37)	7,671	1.84 (1.78–1.90)
2 diseases	7,236	2.06 (2.01–2.11)	3,190	3.37 (3.24–3.52)	2 diseases	5,452	2.15 (2.15–2.27)	2,306	3.55 (3.39–3.72)
3 diseases	1,443	3.05 (2.89–3.22)	747	5.74 (5.32–6.19)	3 diseases	683	3.16 (2.93–3.41)	333	5.71 (5.11–6.37)
4 diseases	179	3.35 (2.90–3.88)	100	6.67 (5.48–8.13)	4 diseases	56	2.28 (1.75–2.96)	32	4.68 (3.30–6.62)

CVD, cardiovascular disease; DM, diabetes mellitus; HTN, hypertension; IHD, ischemic heart diseases, including myocardial infarction; MI, myocardial infarction.
S/T codes in International Classification of Diseases 10th edition measured the disability of each injury diagnosis
[a] Adjusted for age, sex, cigarette smoking, and body mass index

eTable 12. Age-standardized prevalence of cardiometabolic diseases based on Eastern Asia population for the year 2000 from World Population Prospects 2022

Age Group, years	WPP (thousands)	WPP ratio	Cohorts	Number of multimorbidity in ACC cohorts														
				**HTN**	**DM**	**IHD**	**Stroke**	**DM, HTN**	**IHD, HTN**	**IHD, DM**	**Stroke, IHD**	**Stroke, HTN**	**Stroke, DM**	**IHD, DM, HTN**	**Stroke, IHD, HTN**	**Stroke, DM, HTN**	**Stroke, IHD, DM**	**Stroke, DM, IHD, HTN**
40–44	98978.26	0.20	76199	5553	1059	365	45	300	159	14	1	43	6	8	4	4	0	5
45–49	99626.95	0.20	87733	11044	1791	615	100	773	454	44	3	142	5	40	17	28	1	7
50–54	75923.93	0.15	81197	14128	2348	914	169	1284	806	73	9	309	14	89	31	54	0	31
55–59	58022.47	0.12	77407	16014	2801	1189	259	1643	1125	126	15	460	29	189	62	98	6	38
60–64	51669.69	0.10	66022	15478	2639	1464	360	1962	1615	169	17	668	59	338	118	153	4	66
65–69	44215.41	0.09	49783	12879	2051	1552	368	1871	2072	232	49	791	53	398	187	200	6	80
70–74	32867.11	0.07	23942	6156	1015	878	300	991	1197	139	43	657	56	274	210	173	9	94
75–79	21866.97	0.04	6931	2006	302	313	109	225	303	39	14	135	13	210	48	30	2	17
80–84	11741.18	0.02	1686	497	51	102	31	38	84	8	3	30	1	36	7	3	0	6
85–89	5108.12	0.01	596	143	16	42	14	20	31	2	1	5	0	8	3	2	0	1
≥90	1809.27	0.00	180	43	3	11	3	1	7	0	0	4	0	0	0	0	0	0
				**Prevalence of multimorbidity in ACC cohorts (per 1,000 persons)**														
40–44	98978.26	0.20	76199	72.9	13.9	4.79	0.59	3.94	2.09	0.18	0.01	0.56	0.08	0.11	0.05	0.05	0	0.07
45–49	99626.95	0.20	87733	126	20.4	7.01	1.14	8.81	5.17	0.5	0.03	1.62	0.06	0.46	0.19	0.32	0.01	0.08
50–54	75923.93	0.15	81197	174	28.9	11.3	2.08	15.8	9.93	0.9	0.11	3.81	0.17	1.1	0.38	0.66	0	0.38
55–59	58022.47	0.12	77407	207	36.2	15.4	3.35	21.2	14.5	1.63	0.19	5.94	0.38	2.44	0.8	1.27	0.08	0.49
60–64	51669.69	0.10	66022	234	40	22.2	5.45	29.7	24.5	2.56	0.26	10.1	0.89	5.12	1.79	2.32	0.06	1
65–69	44215.41	0.09	49783	259	41.2	31.2	7.39	37.6	41.6	4.66	0.98	15.9	1.06	7.99	3.76	4.02	0.12	1.61
70–74	32867.11	0.07	23942	257	42.4	36.7	12.5	41.4	50	5.81	1.8	27.4	2.34	11.4	8.77	7.23	0.38	3.93
75–79	21866.97	0.04	6931	289	43.6	45.2	15.7	32.5	43.7	5.63	2.02	19.5	1.88	30.3	6.93	4.33	0.29	2.45
80–84	11741.18	0.02	1686	295	30.2	60.5	18.4	22.5	49.8	4.74	1.78	17.8	0.59	21.4	4.15	1.78	0	3.56
85–89	5108.12	0.01	596	240	26.8	70.5	23.5	33.6	52	3.36	1.68	8.39	0	13.4	5.03	3.36	0	1.68
≥90	1809.27	0.00	180	239	16.7	61.1	16.7	5.56	38.9	0	0	22.2	0	0	0	0	0	0
				**Age-standardized prevalence of multimorbidity (per 1,000 persons)**														
40–44	98978.26	0.20	76199	14.4	2.74	0.94	0.12	0.78	0.41	0.04	0	0.11	0.02	0.02	0.01	0.01	0	0.01
45–49	99626.95	0.20	87733	25	4.05	1.39	0.23	1.75	1.03	0.1	0.01	0.32	0.01	0.09	0.04	0.06	0	0.02
50–54	75923.93	0.15	81197	26.3	4.38	1.7	0.32	2.39	1.5	0.14	0.02	0.58	0.03	0.17	0.06	0.1	0	0.06

55–59	58022.47	0.12	77407	23.9	4.18	1.78	0.39	2.45	1.68	0.19	0.02	0.69	0.04	0.28	0.09	0.15	0.01	0.06
60–64	51669.69	0.10	66022	24.1	4.12	2.28	0.56	3.06	2.52	0.26	0.03	1.04	0.09	0.53	0.18	0.24	0.01	0.1
65–69	44215.41	0.09	49783	22.8	3.63	2.75	0.65	3.31	3.67	0.41	0.09	1.4	0.09	0.7	0.33	0.35	0.01	0.14
70–74	32867.11	0.07	23942	16.8	2.78	2.4	0.82	2.71	3.27	0.38	0.12	1.8	0.15	0.75	0.57	0.47	0.02	0.26
75–79	21866.97	0.04	6931	12.6	1.9	1.97	0.68	1.41	1.9	0.24	0.09	0.85	0.08	1.32	0.3	0.19	0.01	0.11
80–84	11741.18	0.02	1686	6.9	0.71	1.42	0.43	0.53	1.17	0.11	0.04	0.42	0.01	0.5	0.1	0.04	0	0.08
85–89	5108.12	0.01	596	2.44	0.27	0.72	0.24	0.34	0.53	0.03	0.02	0.09	0	0.14	0.05	0.03	0	0.02
≥90	1809.27	0.00	180	0.86	0.06	0.22	0.06	0.02	0.14	0	0	0.08	0	0	0	0	0	0

ACC, Asia Cohort Consortium; CVD, cardiovascular disease; DM, diabetes mellitus; HTN, hypertension; IHD, ischemic heart diseases, including myocardial infarction; WPP, World Population Prospects.

eTable 13. Description of the types of effects of cardiometabolic multimorbidity on the risk of all-cause and CVD mortality

Types of effects	Description
Additive effect	is present if the combined effect of two variables is equal to the sum of the individual effects of each variable (A+B=A&B).
Sub-additive effect	is present if the effect of two variables is less than the sum of the individual effects of each variable but greater than the largest individual effect (max (A, B)<A&B<A+B).
Multiplicative effect	is present if the combined effect of two variables is equal to the product of each variable's individual effects (A*B=A&B).
Synergistic effect	is present if the combined effect of two variables is greater than the sum or product of each variable's individual effects (A&B>A+B and A&B>A*B).
Antagonistic effect	is present if the combined effect of the two variables is less than the largest variable effect alone (A&B<max (A, B)).

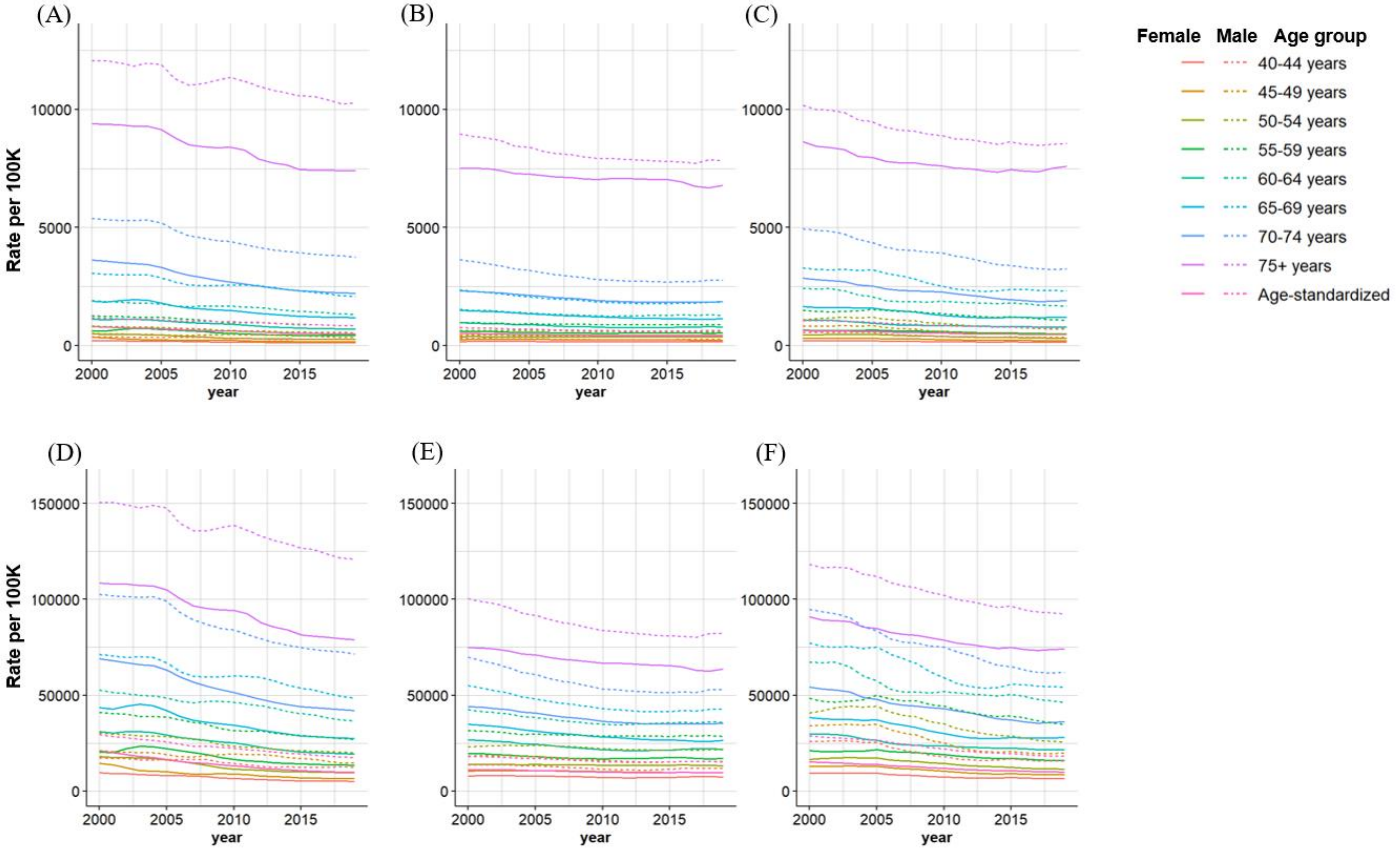

eFigure 1. Death rates and YLLs per 100K for all-cause mortality based on GBD according to region. (**A**) Deaths in East Asia, (**B**) Deaths in North America, (**C**) Deaths in European, (**D**) YLLs in East Asia, (**E**) YLLs in North America, and (**F**) YLLs in European. GBD, Global Burden of Disease (available from https://vizhub.healthdata.org/gbd-results/); YLL, Years of life lost.

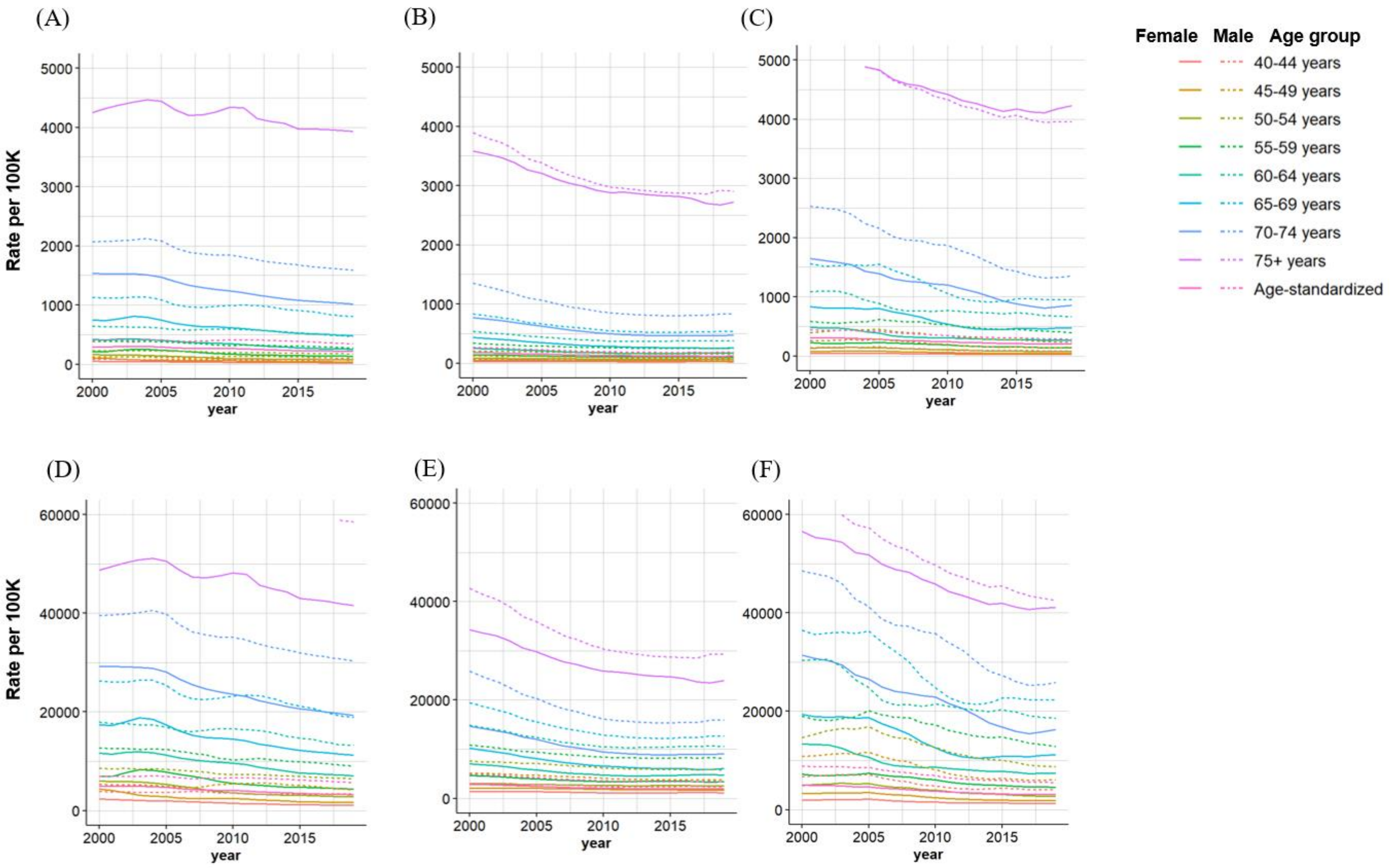

eFigure 2. Death rates and YLLs per 100K for CVD mortality based on GBD according to region. (**A**) Deaths in East Asia, (**B**) Deaths in North America, (**C**) Deaths in European, (**D**) YLLs in East Asia, (**E**) YLLs in North America, and (**F**) YLLs in European. CVD, cardiovascular disease; GBD, Global Burden of Disease (available from https://vizhub.healthdata.org/gbd-results/); YLL, Years of life lost.

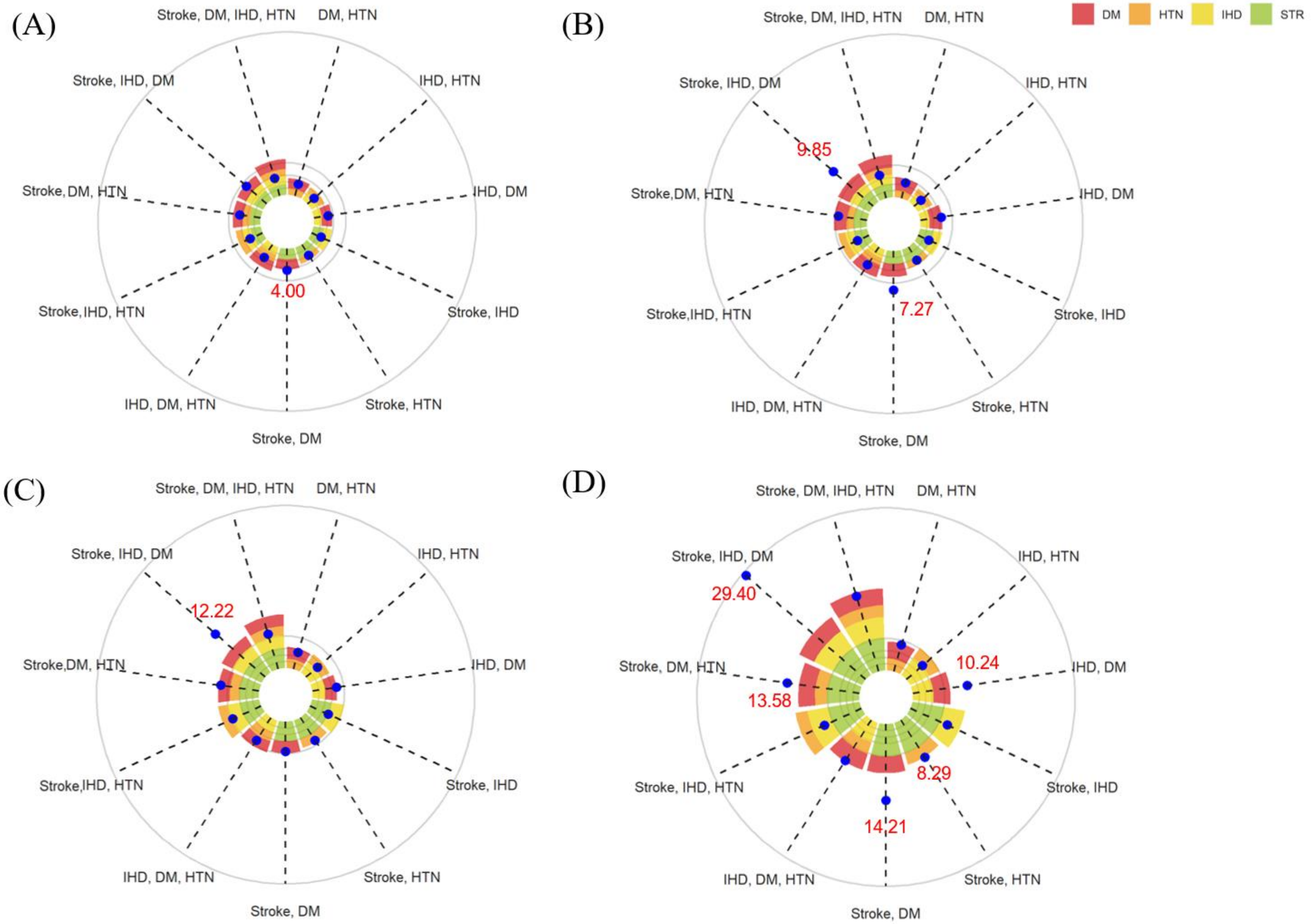

eFigure 3. Risk of each cardiometabolic morbidity on all-cause and cardiovascular disease mortality. The cumulative color bars in circular plot are the expected risks of cardiometabolic multimorbidity associated with individually with either disease, whereas the blue dot is the observed risk of cardiometabolic multimorbidity. (**A**) the risk for all-cause mortality, (**B**) the risk for premature all-cause mortality (age of death <70 in men and women), (**C**) the risk for CVD mortality, (**D**) the risk for premature CVD mortality (age of death <70 in men and women). CVD, cardiovascular disease; DM, diabetes mellitus; HTN, hypertension.
